# Natural and Hybrid Bioadhesive Polymeric Platforms for Bone Regeneration: A PRISMA-Guided Systematic Review of Design Strategies and Translational Challenges

**DOI:** 10.3390/polym18182266

**Published:** 2026-09-17

**Authors:** David Olmos-Villanueva, Karem Noris-Suarez, Miguel Suffo

**Affiliations:** 1 Department of Mechanical Engineering and Industrial Design, High Engineering School, Institute of Research and Biomedical Innovation of Cadiz (INiBICA), Universidad de Cádiz, Campus Río San Pedro s/n, Puerto Real, 11510 Cádiz, Spain; 2 School of Engineering, Science and Technology-Valencia International University (VIU), Calle Pintor Sorolla, 21, 46002 Valencia, Spain

**Keywords:** bioadhesive biomaterials, bone regeneration, wet adhesion, catechol chemistry, systematic review, injectable hydrogels

## Abstract

Bone adhesives have long been pursued in orthopedic and regenerative medicine, but their clinical translation remains limited, particularly in wet and mechanically demanding environments. This PRISMA-guided systematic review analyzes bioadhesive biomaterials and adhesive regenerative platforms for bone regeneration, focusing on material composition, adhesion mechanisms, fabrication strategies, biological performance, and translational limitations. Current evidence shows a clear shift from passive adhesive systems toward multifunctional regenerative platforms that combine wet adhesion with osteogenic, angiogenic, immunomodulatory, antimicrobial, and controlled-release functions. Hybrid polymer networks, catechol-mediated chemistry, Schiff-base reactions, dynamic crosslinking, and nanocomposite or ion-releasing strategies emerge as dominant design approaches. However, adhesion testing remains highly heterogeneous, with lap shear, tensile, peel, and related assays performed under variable dry, wet, or pseudo-physiological conditions, limiting cross-study comparison and benchmarking against clinical standards. In vivo evidence is mainly concentrated in small-animal, non-load-bearing defect models, whereas large-animal validation, load-relevant testing, long-term safety data, and disease-specific models remain scarce. Overall, the main translational bottleneck appears to lie not only in adhesive chemistry, but also in the absence of standardized, bone-specific, and mechanically relevant interfacial performance metrics. This review provides an integrated perspective linking adhesion mechanics, biological function, and translational readiness to guide the future development of bioadhesive biomaterials for bone regeneration.

## 1. Introduction

Bone regeneration remains a major clinical challenge in situations where the intrinsic healing capacity of bone is insufficient to restore structural and functional integrity. The clinical and socioeconomic burden of bone repair is substantial at the global level. According to the Global Burden of Disease 2019 analysis, approximately 178 million new fractures occurred worldwide in 2019, with 455 million prevalent cases of acute or long-term fracture-related symptoms and 25.8 million years lived with disability attributable to fractures [1]. Fragility fractures also represent a growing challenge in aging populations, with up to 37 million fragility fractures estimated annually among individuals older than 55 years [2]. In parallel, oncological bone loss and reconstruction needs remain relevant, as global cancer incidence reached approximately 20 million new cases in 2022, and tumor resections may generate complex skeletal defects requiring reconstructive strategies [3]. Craniofacial reconstruction also represents a substantial burden; for example, orofacial clefts alone accounted for approximately 183,000 incident cases globally in 2021 [4]. Although precise worldwide estimates are not available for every indication, including large segmental defects and revision-related bone loss, these figures illustrate the breadth of clinical scenarios in which improved bone-regenerative and fixation strategies are needed.

Large segmental defects, osteoporotic fractures, tumor resections, revision surgeries, craniofacial defects, and trauma-related bone loss often require biomaterial-assisted repair strategies [5,6,7,8,9]. In these scenarios, successful regeneration depends not only on osteogenesis and tissue integration, but also on achieving stable fixation within a hydrated, biologically active, and mechanically dynamic environment. Current clinical approaches are dominated by metallic fixation systems, autologous and allogeneic bone grafts, polymethyl methacrylate (PMMA) bone cements, calcium phosphate cements, and conventional surgical adhesives. Although autologous bone grafting is still considered a reference strategy in many reconstructive scenarios, its limitations include donor-site morbidity, limited availability, additional surgical time, and variable outcomes in compromised patients [6]. Metallic plates, screws, and fixation devices provide mechanical stability, but they may increase surgical invasiveness, induce stress shielding, require secondary removal procedures, and fail to actively support biological regeneration [10]. PMMA cements offer immediate mechanical support but are biologically inert, non-degradable, and associated with thermal, mechanical, and long-term interfacial complications [11]. Calcium phosphate-based materials provide improved bioactivity and chemical similarity to bone mineral, but their brittleness, low toughness, and limited adhesion under wet physiological conditions restrict their use in mechanically demanding applications [12]. Conventional tissue adhesives, including fibrin-based systems and cyanoacrylates, have also shown limited suitability for bone repair because of insufficient wet adhesion, rapid degradation, cytotoxicity concerns, or inadequate mechanical stability [5,7,13,14,15,16,17].

A central limitation shared by many conventional approaches is their limited ability to establish robust and durable adhesion at the bone–material interface. Bone is not an inert substrate, but a hydrated, porous, mineralized, vascularized, and continuously remodeling tissue, which creates a particularly challenging environment for achieving durable wet adhesion [13]. Its surface chemistry, roughness, porosity, cellular activity, and mechanical environment vary according to anatomical site, defect type, healing stage, and pathological condition [18]. These features make adhesion to bone fundamentally more complex than adhesion to dry engineering substrates [19]. Therefore, generic tissue adhesives and bone-specific bioadhesives should not be considered interchangeable. The former are commonly designed for soft-tissue sealing, wound closure, hemostasis, or temporary tissue approximation, where compliance, rapid sealing, and biocompatibility are often the dominant requirements. In contrast, bone-specific bioadhesives must bond to hydrated and mineralized interfaces, tolerate surface roughness and porosity, resist displacement under mechanically demanding conditions, and ideally support osteogenesis, osseointegration, mineralization, and long-term interfacial remodeling. This distinction is particularly important because adhesive performance demonstrated on soft tissues, glass, polymers, or non-mineralized substrates does not necessarily predict effective bonding to bone or clinically meaningful fixation in orthopedic and craniofacial applications.

In addition, clinically relevant conditions such as osteoporosis, diabetes, infection, inflammation, or impaired vascularization can alter both the biological and mechanical environment of the defect, further compromising interfacial bonding and regenerative outcomes [5,7,9,13]. In response to these limitations, advanced biomaterial platforms, including hydrogels, smart grafts, 3D-printed scaffolds, and stimuli-responsive systems, have been developed to improve structural support, biological performance, and patient-specific regeneration [13,14,15,16,17]. Unlike conventional scaffolds or passive fillers, these systems are designed to interact actively with wet bone surfaces while simultaneously supporting biological repair. Bioadhesive strategies include injectable hydrogels, adhesive scaffolds, guided bone regeneration membranes, surface-functionalized implants, nanocomposite systems, and bioinspired coatings [20,21,22,23,24,25,26,27,28]. Their clinical appeal lies in their potential to conform to irregular defects, improve interfacial integration, reduce surgical invasiveness, deliver bioactive agents locally, and combine fixation with regeneration [13,15,16,20,21].

At the molecular level, wet adhesion is commonly achieved through dynamic covalent, supramolecular, and bioinspired interactions. Schiff-base reactions, hydrogen bonding, electrostatic interactions, metal–ligand coordination, boronate ester formation, and catechol-mediated chemistry are among the most frequently explored mechanisms [13,15,16,20,21]. Catechol-based strategies inspired by mussel adhesive proteins have attracted particular attention because they can promote adhesion to both organic and inorganic substrates under wet conditions, including mineralized and bone-relevant interfaces [20,21,22,24,25,26,28]. These adhesive mechanisms are often integrated with dynamic or dual-crosslinked polymer networks to provide injectability, self-healing capacity, shape adaptation, and mechanical resilience [13,15,16,29]. Beyond adhesion, contemporary bioadhesive biomaterials are increasingly designed as multifunctional regenerative platforms. The incorporation of hydroxyapatite, bioactive glass, calcium- or magnesium-releasing phases, extracellular vesicles, growth-factor-mimetic peptides, antimicrobial agents, antioxidants, and immunomodulatory molecules enables these systems to regulate multiple aspects of the bone healing microenvironment. As a result, current designs aim not only to bond to bone, but also to promote osteogenesis, angiogenesis, macrophage polarization, antibacterial protection, oxidative stress regulation, and controlled therapeutic release [13,15,16,20,21,29]. This evolution reflects a broader transition from simple adhesive materials toward smart, bioactive, and biologically instructive platforms.

Despite this rapid progress, clinical translation remains limited. A major barrier is the absence of standardized evaluation methods for bone-specific wet adhesion. Adhesion performance is commonly assessed using lap-shear, tensile, peel, burst-pressure, or custom-made tests. These assays are performed under highly variable conditions, including dry, wet, simulated body fluid, blood-contact, or pseudo-physiological environments. Differences in substrates, sample preparation, hydration state, loading mode, measurement units, and failure analysis make it difficult to compare reported adhesion strength values across studies. Moreover, many investigations lack benchmarking against clinically established materials such as PMMA, calcium phosphate cements, commercial membranes, or standard fixation approaches [10,11,12,13,20,21]. Another important limitation is the mismatch between promising laboratory performance and clinically relevant validation. Most in vivo studies rely on small-animal, non-load-bearing defect models, particularly rodent calvarial defects, which are useful for proof-of-concept evaluation but do not fully reproduce the mechanical, anatomical, vascular, and immunological complexity of human bone repair. Large-animal studies, long-term implantation data, mechanically loaded models, and disease-specific conditions remain scarce. Consequently, the translational readiness of many bioadhesive systems remains difficult to determine, even when their in vitro and early in vivo outcomes are encouraging [5,7,8,9,13,20]. Therefore, the current challenge in the field is not only to develop stronger or more bioactive adhesives. It is also necessary to integrate interfacial mechanics, biological performance, and translational potential into a coherent strategy for the design and evaluation of bioadhesive biomaterials for bone regeneration. Without standardized, bone-specific, and load-relevant metrics, it remains difficult to identify which material strategies are genuinely superior and which are merely promising under simplified experimental conditions. The main factors currently limiting clinical translation of bone-specific bioadhesive biomaterials are summarized in Table 1.

Accordingly, this PRISMA-guided systematic review aims to provide a comprehensive and structured synthesis of bioadhesive biomaterials and adhesive regenerative platforms for bone regeneration from an interface-engineering perspective, enabling systematic comparison of current strategies, identifying knowledge gaps, and highlighting priorities for future research and clinical translation. Specifically, this review aims to (i) classify the main material platforms and design strategies; (ii) analyze wet adhesion mechanisms and fabrication approaches; (iii) evaluate biological performance, including osteogenic, angiogenic, immunomodulatory, and antibacterial functions; (iv) assess the level of in vitro, ex vivo, and in vivo validation; and (v) identify key methodological, mechanical, and translational barriers that currently limit clinical implementation.

## 2. Materials and Methods

### 2.1. Study Design

This study was designed as a systematic review conducted and reported in accordance with the Preferred Reporting Items for Systematic Reviews and Meta-Analyses 2020 statement (PRISMA 2020) and its explanation and elaboration document [38,39]. The completed PRISMA 2020 checklist is provided as Supplementary Material. A review protocol was prepared in PROSPERO before completion of the review; however, the record was not formally published and no PROSPERO registration number is currently available. The review question and eligibility criteria were structured using the PICOC framework, including Population, Intervention, Comparison, Outcome, and Context, to ensure a transparent definition of the scope and to improve methodological reproducibility.

**Figure 1:**
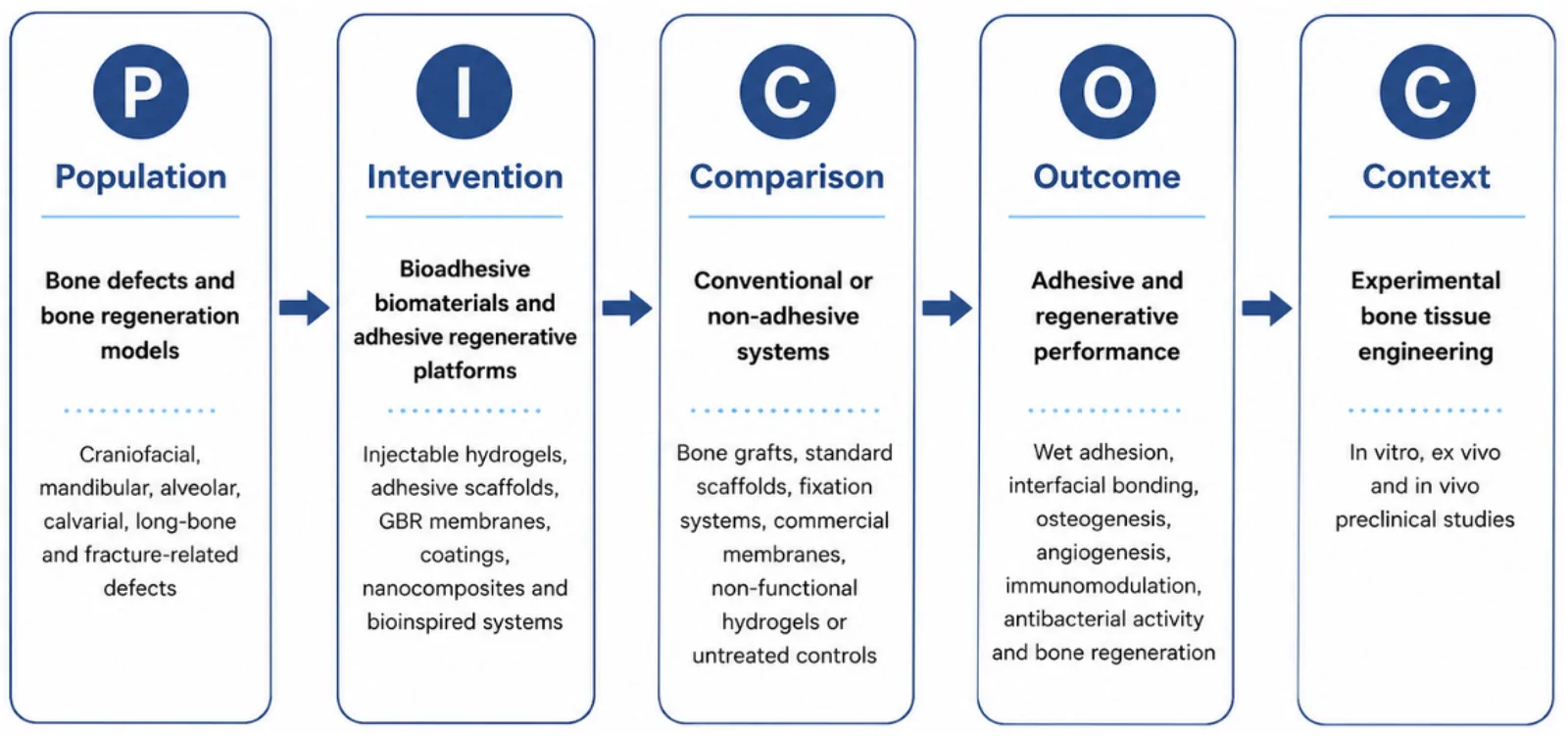
PICOC framework used to define the systematic review question. Abbreviation: GBR membrane, guided bone regeneration membrane.

In this review, the population comprised experimental models of bone defects and bone regeneration, including craniofacial, mandibular, alveolar, calvarial, long-bone, and fracture-related defects. The intervention included bioadhesive biomaterials and adhesive regenerative platforms, such as injectable hydrogels, adhesive scaffolds, guided bone regeneration membranes, functionalized coatings, nanocomposite systems, and bioinspired adhesive materials. Comparators included conventional non-adhesive biomaterials, bone grafts, standard scaffolds, fixation systems, commercial membranes, non-functional hydrogels, or untreated controls when applicable. Outcomes included adhesive performance, wet interfacial bonding, osteogenesis, angiogenesis, immunomodulation, antibacterial activity, cytocompatibility, and in vivo bone regeneration. The context was limited to experimental studies in bone tissue engineering and regenerative medicine performed under in vitro, ex vivo, or in vivo preclinical conditions. The PICOC framework used to define the review question is summarized in Figure 1.

### 2.2. Operational Definitions

For the purpose of this review, the term “bioadhesive” was operationally defined as a biomaterial intentionally designed to establish physical, chemical, or physicochemical bonding with biological tissues under dry or wet conditions. “Bone adhesive” was defined as a bioadhesive material specifically evaluated for bonding, fixation, retention, sealing, coating, or interfacial integration in bone-related substrates, bone defects, bone implants, or bone-regeneration models.

“Adhesive biomaterial” was used as a broader term for materials in which adhesion or interfacial bonding represented a relevant design feature, functional property, or experimental outcome, even when bone was not the only substrate used for mechanical testing. “Adhesive regenerative platform” was defined as an adhesive biomaterial that combined interfacial bonding with at least one bone-regenerative biological function, such as osteogenesis, angiogenesis, immunomodulation, antibacterial activity, hemostasis, osseointegration, or controlled bioactive delivery.

During study selection, these terms were distinguished according to both the intended application and the evidence reported by each study. Studies were considered eligible when they included a clearly described adhesive or interfacial design strategy and a direct bone-related regenerative application.

Studies were considered to meet the minimum adhesive criterion when they reported at least one explicit adhesion-related design feature, adhesion mechanism, or adhesion-related experimental outcome. Eligible evidence included quantitative adhesion testing, such as lap-shear, tensile adhesion, peel, push-out, pull-out, burst pressure, or interfacial toughness assays, as well as clearly described adhesive fixation, coating retention, tissue/material bonding, or interfacial integration relevant to bone-regeneration models.

Qualitative tissue retention, hemostasis, generic cell adhesion, or adhesion to non-bone substrates alone was not considered sufficient for inclusion unless accompanied by a clearly described adhesive mechanism or additional evidence of tissue/material adhesion, interfacial bonding, adhesive fixation, coating retention, or bone-related interfacial integration. Adhesion to non-bone substrates, such as glass, titanium, PMMA, or soft tissue, was considered supportive but not sufficient by itself unless the study also demonstrated direct relevance to bone regeneration, bone repair, osseointegration, guided bone regeneration, or fracture-related healing.

### 2.3. Search Strategy

A comprehensive literature search was performed in four electronic databases: PubMed/MEDLINE, Scopus, Web of Science, and IEEE Xplore. These databases were selected to ensure broad coverage of biomedical, materials science, and engineering literature related to bioadhesive biomaterials, adhesive regenerative platforms, and bone tissue engineering. The search strategy was developed according to the PICOC framework and combined controlled vocabulary, when available, with free-text terms. Search terms were organized into four main conceptual blocks: (i) bioadhesive and interfacial adhesion terms; (ii) biomaterial platform terms; (iii) smart, bioactive, or multifunctional material terms; and (iv) bone regeneration-related terms. The first block included terms such as “bioadhesive”, “tissue adhesive”, “bone adhesive”, “adhesive hydrogel”, “wet adhesive”, “tissue sealant”, “surgical sealant”, “injectable adhesive”, “self-healing hydrogel”, “adhesive scaffold”, “interfacial bonding”, “catechol”, and “mussel-inspired”. The second block included “hydrogel*”, “scaffold*”, “membrane*”, “coating*”, “nanocomposite*”, “composite*”, “hybrid composite”, and “biomaterial*”. The third block included functional descriptors such as “smart”, “multifunctional”, “stimuli-responsive”, “responsive”, “bioactive”, “immunomodulatory”, “osteoimmunomodulatory”, “controlled release”, “antibacterial”, and “angiogenic”. The fourth block included bone-related terms such as “bone regeneration”, “bone tissue engineering”, “bone defect*”, “guided bone regeneration”, “craniofacial bone”, “mandibular bone”, “alveolar bone regeneration”, “osteogenesis”, “bone repair”, “bone healing”, “fracture*”, and “osseointegration”. The search equations were adapted to the syntax and indexing requirements of each database. Boolean operators, truncation symbols, phrase searching, and field tags were used when appropriate to maximize sensitivity while maintaining relevance. Searches were limited to original research articles published in English from January 2016 to May 2026, with the final search performed on 11 May 2026. Non-experimental publications, including reviews, systematic reviews, meta-analyses, editorials, letters, conference abstracts, book chapters, patents, and unpublished studies, were excluded. Studies unrelated to bone regeneration or without a clear adhesive, bioadhesive, or interfacial component were also excluded.

The complete database-specific search strategies are provided in Supplementary Material, Supplementary File S1. For each database, the full search string is reported, including search fields, Boolean operators, truncation symbols, phrase searching, filters, document-type restrictions, language limits, publication-year limits, and the final search date. The final search was performed on 11 May 2026. To evaluate the robustness of the search strategy and reduce the risk of missing eligible studies using alternative terminology, a supplementary sensitivity search was conducted using additional terms suggested for bone-specific adhesive systems. These terms included “bone glue”, “osteoadhesive”, “fracture adhesive”, “mineral-organic adhesive”, “adhesive periosteum”, “adhesive membrane”, and “interfacial toughness”. The sensitivity search was performed in the same databases and within the same date range as the primary search. Retrieved records were checked against the final included dataset and assessed according to the same eligibility criteria. This supplementary search did not identify additional eligible studies beyond those already included in the final qualitative synthesis.

### 2.4. Eligibility Criteria

Studies were considered eligible for inclusion when they met all predefined criteria. These included: (i) original experimental research articles published in peer-reviewed scientific journals; (ii) studies published in English from January 2016 to May 2026, with the final search performed on 11 May 2026; (iii) studies evaluating bioadhesive biomaterials, adhesive regenerative platforms, or materials with a clear interfacial adhesion strategy; (iv) studies focused on bone regeneration, bone tissue engineering, bone repair, guided bone regeneration, osseointegration, or fracture-related healing; (v) studies involving biomaterial platforms such as injectable hydrogels, adhesive scaffolds, functionalized membranes, surface coatings, nanocomposites, bioinspired systems, or composite biomaterials; (vi) studies providing experimental validation using in vitro, ex vivo, and/or in vivo models; and (vii) studies reporting outcomes related to adhesive performance, wet interfacial bonding, osteogenic activity, angiogenesis, immunomodulation, antibacterial activity, cytocompatibility, osseointegration, or bone regeneration. Studies were excluded when they met any of the following criteria: (i) publications written in languages other than English; (ii) non-experimental publications, including narrative reviews, systematic reviews, meta-analyses, expert opinions, editorials, letters to the editor, conference abstracts, case reports, case series, book chapters, patents, and unpublished studies; (iii) studies not published in peer-reviewed journals; (iv) studies focused exclusively on non-bone tissues, such as skin, cartilage, tendon, vascular, neural, or general soft-tissue repair; (v) studies evaluating biomaterials without a clear adhesive, bioadhesive, interfacial, or surface-functionalization strategy; (vi) studies without direct relevance to bone regeneration, bone repair, guided bone regeneration, osseointegration, or fracture healing; and (vii) studies not reporting experimental outcomes related to adhesion, biological performance, or bone-regenerative activity.

### 2.5. Study Selection

**Figure 2:**
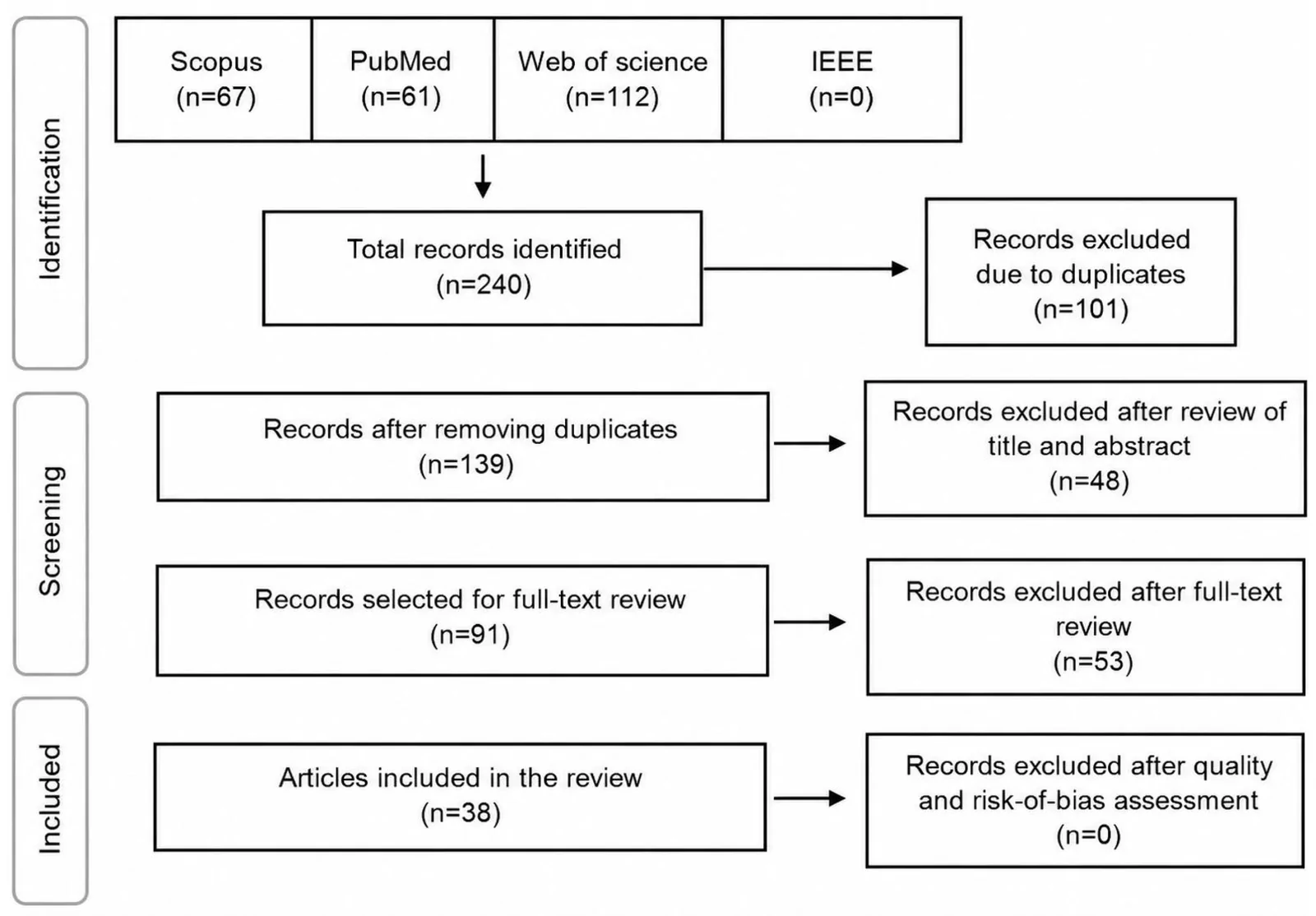
PRISMA 2020 flow diagram showing the identification, screening, eligibility assessment, and inclusion of studies through databases and registers. Reasons for full-text exclusion are provided in Supplementary Table S3.

All records retrieved from the selected databases were exported and compiled in Parsifal, a web-based tool for managing systematic literature reviews. Duplicate records were identified and removed before screening. The remaining records were screened by title and abstract according to the predefined eligibility criteria. Screening decisions were assigned within Parsifal using the predefined inclusion and exclusion criteria, and records were classified as accepted, rejected, duplicated, or unclassified during the selection process. Records considered potentially eligible after title and abstract screening were retrieved for full-text assessment. Full-text articles were then evaluated against the inclusion and exclusion criteria described above. Studies were included when they addressed both an adhesive, bioadhesive, interfacial, or surface-functionalization strategy and a direct application in bone regeneration, bone repair, guided bone regeneration, osseointegration, or fracture-related healing. Uncertain cases were discussed among the review team until consensus was reached. When eligibility could not be determined from the title and abstract alone, the article was retained for full-text assessment. The complete selection process, including the number of records identified, duplicates removed, records screened, full-text articles assessed, and studies included in the final qualitative synthesis, is summarized in the PRISMA 2020 flow diagram shown in Figure 2.

A total of 240 records were initially identified through database searching. After duplicate removal, 139 records were screened by title and abstract. Of these, 48 records were excluded because they did not meet the predefined eligibility criteria. The remaining 91 records were selected for full-text assessment. After full-text review, 53 articles were excluded for not meeting the inclusion criteria or for insufficient relevance to bone bioadhesive biomaterials. Finally, 38 studies met all eligibility criteria and were included in the qualitative synthesis. The full-text eligibility assessment, including included studies, excluded studies, and reasons for exclusion, is provided in Supplementary Table S3.

### 2.6. Data Extraction

Data extraction was performed independently by two reviewers using a structured and standardized form specifically designed for this review. Before full extraction, the form was piloted on a subset of included studies to ensure consistency in the interpretation of the extraction variables. Extracted data were then compared between reviewers. Discrepancies were resolved through discussion and consensus, and when agreement could not be reached, a third reviewer was consulted. The final extraction matrix was reviewed by all authors before qualitative synthesis. The extraction framework was developed to capture bibliographic, methodological, material, adhesive, biological, and translational information from each included study. The main data extraction domains and variables are summarized in Table 2. This approach allowed comparison across heterogeneous bioadhesive biomaterials and adhesive regenerative platforms for bone regeneration. For each included article, general study characteristics were recorded, including author, year of publication, journal, study design, experimental model, bone application, anatomical site, defect type, comparator or control group, and type of validation performed. When available, information regarding sample size, animal species, duration of follow-up, and ethical approval was also extracted. Material-related data included the biomaterial platform, polymeric or inorganic composition, natural or synthetic origin of the main components, presence of bioinspired motifs, incorporation of catechol- or mussel-inspired chemistry, nanocomposite design, and use of bioactive agents such as hydroxyapatite, bioactive glass, metallic ions, extracellular vesicles, peptides, growth factors, antimicrobial agents, antioxidants, or immunomodulatory molecules. Fabrication and handling characteristics were also extracted, including injectability, in situ gelation, self-healing capacity, stimulus-responsiveness, crosslinking strategy, degradation behavior, and controlled release properties.

Adhesive and mechanical performance was recorded by extracting the reported adhesion mechanism, testing environment, substrate used, type of adhesion assay, and quantitative adhesion values when available. Particular attention was paid to whether adhesion was evaluated under dry, wet, simulated physiological, blood-contact, or bone-relevant conditions. Additional mechanical parameters, such as compressive strength, tensile strength, elastic modulus, toughness, fatigue resistance, or fixation performance, were extracted when reported. Biological outcomes included cytocompatibility, cell adhesion and spreading, proliferation, osteogenic differentiation, mineralization, angiogenic activity, immunomodulatory effects, macrophage polarization, inflammatory cytokine modulation, antibacterial activity, and oxidative stress regulation. In vivo outcomes were extracted when available, including animal model, defect location, defect size, bone formation, osseointegration, vascularization, degradation, safety, local inflammatory response, and comparison with clinically used materials.

Finally, translational indicators were recorded, including evidence of load-bearing or mechanically relevant testing, large-animal validation, long-term follow-up, scalability, manufacturing considerations, sterilization or storage issues, regulatory discussion, toxicity concerns, adverse effects, and authors’ stated limitations. Extracted information was organized into comparative tables to support qualitative synthesis across material design, adhesion mechanisms, biological performance, and translational readiness.

### 2.7. Data Synthesis

Due to the expected heterogeneity of the included studies in terms of material composition, biomaterial platform, adhesion mechanism, fabrication strategy, biological evaluation, and in vivo model, a quantitative meta-analysis was not performed. Differences in experimental design, testing substrates, adhesion assays, mechanical loading conditions, biological endpoints, animal models, and reporting units were considered too substantial to allow meaningful pooled quantitative analysis. Therefore, a qualitative evidence synthesis was conducted. The included studies were synthesized according to the predefined extraction and classification criteria summarized in Table 2, Table 3, Table 4 and Table 5. Material-related information was first extracted according to the domains summarized in Table 2 and then classified according to primary polymeric matrix (Table 3), structural reinforcement strategy (Table 4), and incorporation of bioactive cargos (Table 5). This classification enabled comparison of natural, synthetic, bioinspired, and hybrid polymeric systems, together with the use of inorganic reinforcing phases, therapeutic molecules, extracellular vesicles, growth factors, and other functional additives. Subsequently, studies were comparatively analyzed according to their adhesion mechanisms, crosslinking strategies, mechanical characterization, biological performance, and translational potential. Adhesive performance was evaluated by considering the dominant interfacial bonding mechanisms, testing methodologies, experimental conditions, and quantitative mechanical outcomes whenever reported. Biological performance was synthesized according to the principal regenerative functions investigated, including osteogenesis, mineralization, angiogenesis, immunomodulation, antibacterial activity, oxidative stress regulation, cytocompatibility, osseointegration, and in vivo bone regeneration, while multifunctionality was identified when adhesive systems simultaneously integrated mechanical fixation with two or more additional therapeutic or regenerative functions.

Finally, translational readiness was assessed qualitatively by considering the type of validation performed, including in vitro, ex vivo, and in vivo evidence; the use of small- or large-animal models; defect type and anatomical location; follow-up duration; evaluation under mechanically relevant conditions; comparison with clinically used materials; degradation and safety data; scalability; and discussion of regulatory or manufacturing aspects. The synthesis was used to identify dominant design trends, recurring methodological limitations, and key translational gaps in bioadhesive biomaterials for bone regeneration.

## 3. Results

### 3.1. Clinical Landscape of Bone Fixation Materials and the Transition Toward Multifunctional Bone Bioadhesives

**Figure 3:**
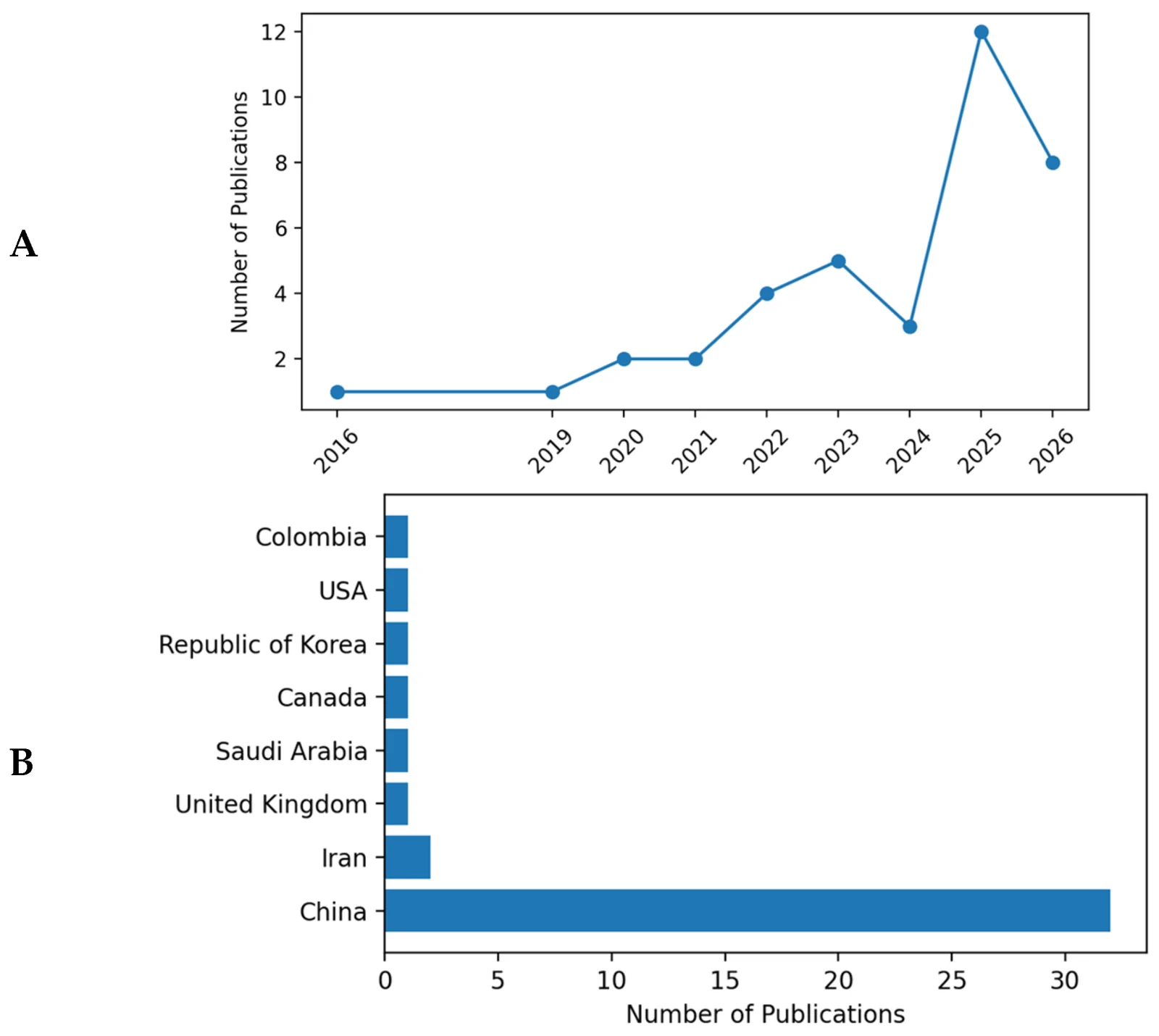
(**A**) Temporal trend of publications on bone adhesives (2016–May 2026). (**B**) Geographical distribution of included studies by country of origin.

The temporal evolution and geographical distribution of the included studies provide essential context for understanding the current state of bone adhesive research. As shown in Figure 3A, the number of publications has experienced a remarkable surge, particularly from 2021 onwards, with a peak of 12 publications in 2025 and an already notable count of 8 in 2026 (as of the data cut-off). This upward trend indicates a growing and accelerating interest in this field over the last five years. Furthermore, Figure 3B shows the global distribution of these studies, revealing a strong geographic concentration. China dominates the landscape with 32 published studies, far surpassing other countries, while contributions from nations such as Iran (2), the United Kingdom (1), the USA (1), and others remain limited. This distribution suggests that research activity is primarily driven by a few key regions, with China emerging as the undisputed leader in this area.

The current research landscape in bone adhesives reveals a marked transition in material design, moving away from conventional approaches toward increasingly complex and functionally integrated systems. This paradigm shift is clearly reflected in the distribution of studies included in this systematic review, which have been classified into four main categories according to their level of technological sophistication and biological functionality.

To improve reproducibility, explicit operational criteria were applied to assign each included study to one developmental category. Type I was defined as a conventional adhesive or fixation system primarily designed to bond, seal, or mechanically stabilize tissue or bone without demonstrated intrinsic bone-regenerative activity. Type II was defined as a bioactive or regenerative adhesive combining an adhesive or interfacial function with one principal bone-regenerative function, most commonly osteogenesis, osseointegration, osteoconduction, mineralization, or bioactive ion release. Type III was defined as a multifunctional regenerative platform combining adhesion with at least two additional therapeutic or regenerative functions, such as osteogenesis, angiogenesis, immunomodulation, antibacterial activity, hemostasis, antioxidant activity, or controlled delivery. Type IV was defined as a smart multifunctional platform combining adhesive performance and regenerative multifunctionality with stimulus-responsive, adaptive, or dynamically regulated behavior, including responsiveness to pH, reactive oxygen species, enzymes, light, metabolic cues, or other microenvironmental triggers.

Each study was classified by evaluating the reported material design, adhesive mechanism, biological functions, and responsive or adaptive features. When a study fulfilled criteria for more than one category, it was assigned to the highest category supported by the experimental evidence. Classification was based on reported experimental data rather than on terminology used by the original authors. The final category assigned to each included study was reviewed by the authors and is reported in Supplementary Table S1.

Type I was retained in the developmental framework as a conceptual baseline rather than as an eligible study category represented in the final dataset. Its inclusion allows the proposed classification to illustrate the transition from conventional adhesive or fixation systems toward bioactive, multifunctional, and smart regenerative adhesive platforms. However, no Type I systems were included among the 38 eligible studies because the inclusion criteria required both an adhesive or interfacial strategy and direct bone-regenerative relevance.

**Figure 4:**
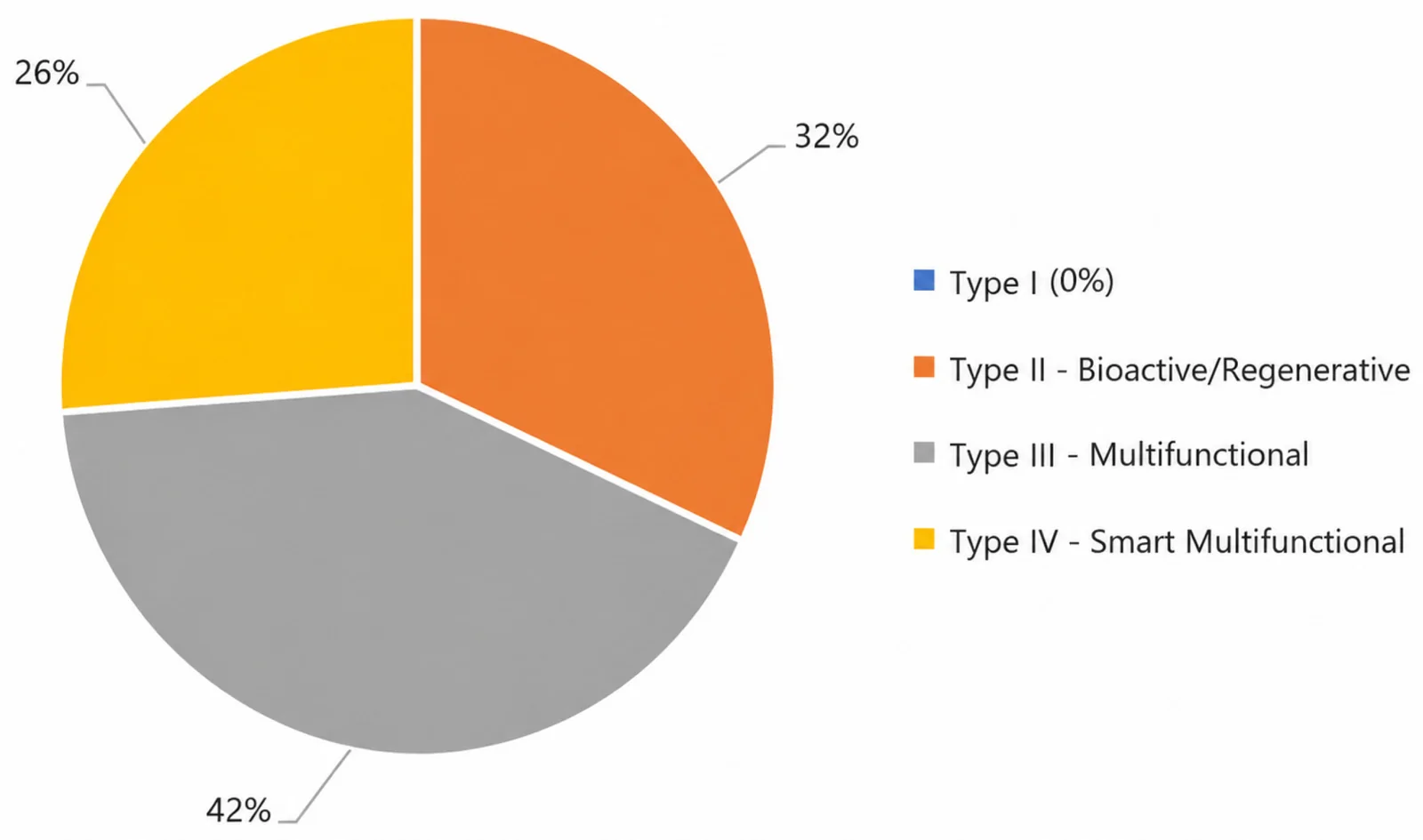
Developmental hierarchy of bone bioadhesive designs according to functional and translational complexity. Type I represents conventional adhesive or fixation systems and is retained as a conceptual baseline. However, no Type I systems were identified among the eligible studies because inclusion required both an adhesive or interfacial strategy and direct bone-regenerative relevance.

As shown in Figure 4, the proposed developmental framework comprised four categories, although the eligible dataset was represented only by Types II–IV: (I) conventional adhesive systems, designed primarily to bond, fix, or seal tissues without intrinsic regenerative activity; (II) bioactive/regenerative adhesives, which combine adhesion with demonstrated bone-regenerative potential; (III) multifunctional regenerative platforms, which integrate adhesion with at least two additional therapeutic or regenerative functions; and (IV) smart multifunctional platforms, which further incorporate stimulus-responsive behavior alongside regenerative functionality. The eligibility criteria specifically targeted bioadhesive materials designed for bone regeneration, resulting in a dataset composed exclusively of biologically functional adhesive systems. Consequently, conventional fixation materials (Type I, 0%) were not represented within the included literature, as they generally fall outside the scope of regenerative bioadhesive research.

In contrast, 32% of the studies are classified as bioactive or regenerative adhesives (Type II). This category includes systems that, while not yet achieving the complexity of more advanced designs, already incorporate essential biological properties such as osteoconduction, controlled ion release, or partial biodegradability. These materials represent a significant step beyond inert approaches, facilitating more active integration with the host bone and establishing the foundation for tissue regeneration. The majority of research, however, is concentrated in the multifunctional adhesive category (Type III), comprising 42% of the studies. These systems are no longer conceived merely as structural fixators but as regenerative platforms that actively orchestrate the healing microenvironment. Their designs integrate capabilities such as immune response modulation, angiogenesis promotion, antibacterial activity, and localized delivery of therapeutic agents, all while providing adequate initial fixation and enabling degradation synchronized with new bone formation. Finally, 26% of the studies represent the cutting edge of the field: smart multifunctional adhesives (Type IV). This category includes systems that incorporate stimuli-responsive materials (responsive to changes in pH, temperature, or enzymatic activity) or employ dynamic covalent and supramolecular crosslinking strategies to adapt their mechanical properties and degradation profiles to the changing needs of the regenerative process. These advanced designs most closely approximate the ideal of a biomaterial that not only fixes bone but actively participates in and adapts to the various phases of bone healing.

Collectively, this classification demonstrates that bone adhesive design has drastically evolved toward increasingly complex and biologically interactive systems, moving beyond passive approaches. The high percentage of multifunctional systems and the emergence of smart platforms reflect the growing convergence between materials science, bone biology, and tissue engineering, all aimed at addressing the persistent unmet clinical need for an adhesive that combines mechanical fixation with active tissue regeneration. For transparency and reproducibility, the complete classification of all included studies according to the proposed developmental framework (Types I–IV) is provided in Supplementary Table S1.

### 3.2. Material-Based Classification

The material landscape of bone bioadhesives is characterized by remarkable compositional diversity, reflecting the convergence of polymer chemistry, materials science, nanotechnology, and regenerative medicine. Rather than converging toward a single optimal material platform, the included studies reveal a progressive evolution toward hierarchically engineered multifunctional systems in which material composition is organized around distinct yet complementary design layers. These platforms generally consist of a structural polymeric matrix that provides an adhesive framework, frequently reinforced with inorganic nanomaterials to enhance mechanical performance and osteoconductivity, and increasingly supplemented with therapeutic cargos capable of actively modulating the biological microenvironment.

Polymer categories were not treated as mutually exclusive. Because many bone bioadhesive systems were formulated as hybrid or interpenetrating networks, a single study could include more than one polymeric component and therefore contribute to more than one category. Accordingly, Table 3 reports the presence of relevant polymeric matrices across the included studies rather than assigning each study to a single exclusive material class.

Among the 38 studies included in this review, natural polymers clearly dominated the material design, as summarized in Table 3. Gelatin-based systems were the most frequently employed (*n* = 12), followed by chitosan (*n* = 8), polyethylene glycol (PEG) (*n* = 6), silk fibroin (*n* = 4), alginate (*n* = 3), dextran-containing formulations (*n* = 4), hyaluronic acid (*n* = 2), polyurethane (*n* = 2), and collagen (*n* = 1). Only a limited number of systems relied predominantly on fully synthetic polymeric networks, highlighting the preference for naturally derived biomaterials because of their intrinsic biocompatibility, biodegradability, extracellular matrix mimicry, and favorable cell–material interactions.

Structural reinforcement represented another major design feature, although it was not universally adopted. The main reinforcement strategies identified across the included studies are summarized in Table 4. Approximately three-quarters of the reported formulations (28/38, 73.7%) incorporated a reinforcing phase, most commonly hydroxyapatite-based nanoparticles, bioactive glass, laponite nanosheets, mesoporous silica nanoparticles, MXene nanosheets, ZIF-8, graphene oxide, black phosphorus nanosheets, or bioactive ceramic particles. These inorganic components primarily served to improve mechanical strength, fracture fixation, and osteoconductivity while simultaneously providing bioactive ionic release. Conversely, 10 studies (26.3%) intentionally omitted reinforcing materials, demonstrating that satisfactory adhesive and regenerative performance can also be achieved through advanced polymer network engineering without introducing inorganic fillers.

A similar trend was observed for therapeutic functionalization, as summarized in Table 5. Approximately two-thirds of the studies (24/38, 63.2%) incorporated bioactive cargos, including growth factors (BMP-2), extracellular vesicles, melatonin-loaded nanovesicles, antibiotics (vancomycin), flavonoids (dihydromyricetin and rutin), antiresorptive drugs (alendronate), anticancer agents (doxorubicin), β-hydroxybutyrate, cell-free fat extracts, antimicrobial silver ions, and osteogenic ions such as Ca^2+^, Sr^2+^, Mg^2+^, and phosphate. In contrast, 14 studies (36.8%) relied exclusively on the intrinsic bioactivity of the material composition, particularly through ion-releasing nanofillers or bioinspired adhesive chemistries, without incorporating exogenous therapeutic molecules.

Overall, these findings suggest that contemporary bone bioadhesives are evolving through two complementary design strategies. The first combines structural polymers with inorganic reinforcing phases to improve mechanical stability while providing osteogenic or angiogenic cues through controlled ion release. Representative examples include the collagen/tetra-PEG bioadhesive reinforced with aminated laponite nanoclay (ALAP) [22] and the BP@TA-Mg-reinforced methacrylated silk fibroin hydrogel reported by Huang et al. [37], both of which combine mechanical reinforcement with bioactive stimulation. The second strategy prioritizes molecular functionalization of the polymeric matrix through therapeutic cargos or bioactive molecules without necessarily increasing structural complexity. Examples include melatonin-loaded mesenchymal stem cell-derived nanovesicles within PEG hydrogels [53] and osteogenic extracellular vesicle-functionalized hyaluronic acid/PEG adhesives [23]. Rather than representing competing approaches, these strategies illustrate the versatility of current bone bioadhesives, which increasingly integrate mechanical stabilization with biologically active tissue regeneration according to specific clinical requirements.

### 3.3. Adhesive, Crosslinking, and Fabrication Strategies

To ensure consistent interpretation, adhesive and crosslinking mechanisms were classified according to the dominant chemical or physicochemical interaction responsible for network formation, interfacial bonding, or cohesion of the adhesive matrix. Because many bioadhesive systems rely on multiple simultaneous interactions, these categories were not considered mutually exclusive. When more than one mechanism was present, the mechanism most directly linked to adhesion, gelation, or interfacial stabilization was identified as the predominant mechanism, while secondary mechanisms were considered supportive.

Supramolecular crosslinking was defined as network formation mediated primarily by reversible non-covalent interactions. These included hydrogen bonding, host–guest interactions, hydrophobic interactions, π–π stacking, electrostatic interactions, and multiple weak cooperative interactions. Coordination crosslinking was defined as network formation or interfacial bonding mediated by metal–ligand interactions, such as catechol–metal complexes or ionic coordination involving biologically relevant ions. Dynamic covalent crosslinking was defined as network formation based on reversible covalent bonds capable of exchange, rearrangement, or self-healing under physiological or near-physiological conditions, including Schiff-base bonds, boronate ester bonds, disulfide exchange, acylhydrazone bonds, or imine-type chemistry. Photochemical crosslinking was defined as light-triggered covalent network formation, typically mediated by photoactive groups or photoinitiators, resulting in polymerization, methacrylate crosslinking, thiol–ene reactions, or photocoupling reactions. Therefore, the classification distinguishes the dominant mechanism of network formation or adhesion rather than the complete chemical complexity of each formulation.

The fabrication approaches reported across the included studies reveal a clear preference for processing strategies compatible with clinical handling while preserving the functional properties of the adhesive materials. Injectable formulations constitute a predominant design strategy, particularly in hydrogel-based and in situ-forming adhesive systems [25,27,28,40,42,47,53]. These formulations are frequently complemented by additional manufacturing or functionalization strategies, including photopolymerization, dynamic covalent crosslinking, nanocomposite reinforcement, surface coating, mineralization, and bioactive cargo incorporation [22,25,28,30,32,35,43,60]. Considerable variability is observed in fabrication methodologies, reflecting the diversity of material compositions and intended clinical applications [22,24,25,26,27,28,30]. Rather than converging toward a single manufacturing route, current research explores multiple processing strategies to optimize handling characteristics, adhesion, and regenerative potential [25,28,30,32,35,42].

Crosslinking chemistry emerged as one of the most consistent design principles across the included studies. Rather than relying on a single gelation mechanism, many reported bioadhesives combined two or more crosslinking strategies, indicating a transition toward hybrid network architectures [22,25,28,30,32,35,40,42,56]. Supramolecular interactions, coordination crosslinking, dynamic covalent bonds such as Schiff-base or boronate chemistry, and photopolymerization were frequently incorporated either independently or in combination [25,28,30,32,35,40,42,55,56]. Instead of acting independently, these mechanisms provide complementary functions. Photopolymerization can supply rapid in situ solidification and early mechanical stability [25,55,56], dynamic covalent bonds can enable injectability, stress relaxation, and self-healing [28,35,40,42], while supramolecular and coordination interactions can dissipate mechanical energy, improve wet adhesion, and increase toughness [22,24,25,26,28,30,32]. Consequently, crosslinking chemistry has evolved from a simple gelation strategy into a modular design tool that allows simultaneous optimization of handling properties, mechanical performance, and biological functionality.

### 3.4. Adhesion Mechanism Under Wet Physiological Conditions

The adhesion-related data demonstrate that achieving stable bonding under physiological wet conditions has become a primary design objective in contemporary bone bioadhesives [20,21,24,25]. Nearly all included studies evaluated adhesion in clinically relevant environments, including wet physiological conditions, simulated body fluid (SBF), and, in several cases, blood-contact interfaces [24,25,35], whereas only a minority relied exclusively on dry testing [52,54,58]. Lap-shear testing was by far the most frequently employed method, often complemented by tensile adhesion, peel, burst pressure, push-out, or other mechanical assays to provide a broader assessment of interfacial performance [24,25,27,30,56]. This distinction between generic tissue adhesion and bone-specific bioadhesion is relevant because adhesion to bone cannot be inferred solely from generic tissue-adhesion assays; mineralized substrates, hydrated interfaces, defect geometry, and mechanical loading conditions strongly influence interfacial bonding and failure behavior [13,19,20,21].

Rather than relying on a single bonding strategy, most bioadhesives combined multiple complementary adhesion mechanisms, including covalent coupling, Schiff-base formation, hydrogen bonding, electrostatic interactions, catechol-mediated chemistry, metal–phenolic coordination, and other supramolecular interactions, to maximize adhesion under hydrated conditions [22,24,25,30,32,42]. Catechol-inspired systems are frequently combined with metal coordination, hydrogen bonding, electrostatic interactions, or dynamic Schiff-base bonds, resulting in multimodal adhesion capable of maintaining stable fixation in the wet and dynamic environment of bone tissue [24,25,28,30,34,42], as reported in representative bone-specific adhesive platforms, including mineral-organic adhesives, mussel-inspired nanocomposite hydrogels, dual-biomimetic bone adhesives, and bisphosphonated injectable adhesives [22,24,25,28,30,34,42]. This cooperative design suggests that optimizing adhesion is not simply a matter of incorporating catechol groups, but rather of integrating complementary mechanisms that act synergistically to maximize adhesive performance [20,21,24,28].

Although quantitative adhesion strength was reported in most studies, the wide variation in testing protocols limits direct comparison between materials [24,25,28,30]. Reported lap-shear values ranged from approximately 9 kPa to nearly 923 kPa, while experimental substrates included bone, skin, glass, titanium, PMMA, mineralized substrates, and polymeric materials [24,26,27,30]. In addition, reported measurement units varied across studies, including kPa, MPa, N, and J/m^2^, further limiting direct benchmarking [27,28,30,56]. Furthermore, a subset of studies did not report quantitative adhesion values, instead reporting hemostatic parameters, rheological properties, or only qualitative descriptions [25,37,43,47]. Collectively, these findings indicate that current research has shifted from optimizing individual adhesive chemistries toward integrating multiple synergistic interactions capable of maintaining robust fixation under clinically relevant wet environments. A detailed summary of the predominant bioadhesion mechanisms identified in the included studies is provided in Supplementary Table S2.

### 3.5. Biological and Functional Performance

Beyond adhesive performance, the included studies consistently demonstrate that contemporary bone bioadhesives are designed as multifunctional regenerative platforms rather than simple fixation materials. Osteogenic performance was evaluated in virtually all included studies, commonly through the combined analysis of osteogenic markers (e.g., ALP, RUNX2, COL1, OCN, OPN and BMP-2), mineralization assays, histological evaluation, and micro-computed tomography (micro-CT), reflecting the central role of bone regeneration in the biological assessment of these systems. In addition to osteogenesis, a substantial proportion of studies investigated complementary biological functions, particularly angiogenesis (through evaluation of markers such as CD31 and VEGF) [23,24,25,32,33,40,45,53] and immunomodulation (through macrophage polarization and inflammatory cytokine analysis) [32,34,35,37,41,46]. Antibacterial activity was also frequently incorporated, primarily against Staphylococcus aureus and Escherichia coli [22,26,42,43,46,47,54], highlighting the increasing interest in preventing implant-associated infections while promoting tissue repair. Hemostatic performance—evaluated through bleeding time and blood loss—was reported in several studies [24,32,33,34,36,37,43,48,53], underscoring the importance of immediate hemorrhage control in trauma-related bone repair. The most recent studies demonstrate a clear transition from purely adhesive materials toward multifunctional therapeutic platforms [25,32,34,35,36,45,59]. Beyond providing stable tissue fixation, many formulations incorporate osteogenic factors, extracellular vesicles, bioactive peptides, flavonoids, antibiotics, or small bioactive molecules capable of modulating inflammation and promoting bone regeneration. At the same time, an increasing number of systems have been engineered to respond to specific microenvironmental cues, including reactive oxygen species (ROS), pH, enzymes, or near-infrared (NIR) irradiation, enabling controlled and on-demand release of therapeutic cargo [25,30,35,37]. Although these smart functionalities are still emerging, they illustrate the direction in which the field is evolving—from passive tissue adhesives to bioresponsive regenerative platforms that actively participate in the healing process [23,35,37,59].

Despite this broad biological characterization, the selection of endpoints, biomarkers, and experimental methodologies remains highly heterogeneous across studies, hindering direct comparison of biological performance. The collected evidence suggests that engineering performance and biological functionality are not always evaluated with the same level of rigor or integration. Many materials are intentionally designed to combine strong adhesion with regenerative activity, yet the balance between these objectives varies considerably among studies. In several cases, extensive characterization of one aspect is accompanied by comparatively limited evaluation of the other, making comprehensive assessment difficult. For instance, while some bioadhesives present detailed mechanical data—including lap-shear and tensile adhesion values under various wet conditions, they provide only qualitative or preliminary biological assessments [24,30,49,55,56,61]. Conversely, other studies emphasize osteogenic differentiation and angiogenic potential through extensive in vitro and in vivo assays but report adhesive performance using non-standardized methods or omit quantitative adhesion strength altogether [34,45,59], as observed in studies reporting only hemostatic parameters or rheological properties [25,37,43]. This apparent mismatch reflects the multidisciplinary nature of bone bioadhesive development and emphasizes the importance of integrating material engineering with biological validation throughout future research.

### 3.6. Mechanical Validation and Experimental Limitations

Mechanical characterization represents an essential component of evaluating bone bioadhesives; however, the extracted data revealed substantial variability in testing methodologies, experimental conditions, and reported outcome measures. Although most studies included quantitative mechanical characterization, no standardized testing protocol was identified across the literature. Adhesive performance metrics were interpreted according to the mechanical meaning of each parameter rather than as interchangeable outcomes. Adhesion strength was defined as the stress required to detach the adhesive interface, usually reported as force normalized by bonded area. Maximum force referred to the peak load recorded during detachment or failure, without necessarily accounting for bonded area. Work of adhesion was defined as the energy required to separate the interface, commonly derived from the area under the force–displacement curve. Interfacial toughness referred to the resistance of the bonded interface to crack initiation or propagation and was considered particularly relevant when fracture-mechanics-based tests were reported. Fixation strength was used for constructs in which the adhesive contributed to the mechanical retention or stabilization of bone fragments, implants, scaffolds, or coatings, and therefore reflected construct-level performance rather than purely interfacial adhesion. Because these parameters depend strongly on test geometry, substrate, hydration state, loading mode, bonded area, curing conditions, and failure mode, values reported across studies were interpreted qualitatively and were not pooled or directly compared as equivalent measures.

In addition, interfacial adhesion was distinguished from bulk mechanical strength and fracture fixation. Interfacial adhesion refers specifically to the ability of the material to bond to a tissue or substrate interface and is typically assessed using lap-shear, tensile adhesion, peel, push-out, pull-out, burst pressure, or interfacial toughness tests. Bulk mechanical strength describes the intrinsic resistance of the material itself to deformation or failure, including compressive strength, tensile strength, elastic modulus, toughness, or fatigue behavior, and does not necessarily indicate effective bonding to bone. Fracture fixation refers to the construct-level capacity of an adhesive system to stabilize bone fragments, implants, coatings, or scaffolds under clinically relevant mechanical conditions. Therefore, strong bulk mechanical properties or improved fracture stability were not interpreted as evidence of interfacial adhesion unless direct bonding or adhesive fixation at the material–bone interface was experimentally demonstrated.

For the purpose of this review, “load-bearing” testing was defined as mechanical evaluation intended to reproduce or approximate the functional loading experienced by bone. This included loading conditions related to fracture stabilization, defect repair, implant fixation, or orthopedic reconstruction, as well as experimental setups assessing the ability of an adhesive system to resist compressive, tensile, bending, torsional, shear, cyclic, or push-out loads applied to bone fragments, bone–implant interfaces, scaffolds, coatings, or defect constructs. “Mechanically relevant” testing was used as a broader term referring to assays that, although not fully reproducing physiological load-bearing conditions, evaluated adhesive or construct performance under clinically meaningful mechanical constraints, such as hydrated testing environments, bone-like or mineralized substrates, fracture or defect geometries, fixation stability, resistance to displacement, or comparison with clinically used materials. Simple rheological measurements, compressive testing of isolated hydrogels, or adhesion testing on non-bone substrates were not considered load-bearing by themselves unless they were linked to a bone-relevant construct, fixation model, or clinically oriented mechanical scenario.

Lap shear was by far the most frequently employed adhesion assay, accounting for nearly three-quarters of the included studies, whereas tensile adhesion was the second most common mechanical test. Other methodologies, including peel testing [30,56], compression [25,26,27,30,31,41,61], push-out [24,30,49,56,60], three-point bending [25,37], wound-closure assays, burst-pressure-related testing, and rheological characterization [25,35,60], were used less frequently and generally reflected the specific clinical application or intended function of the adhesive rather than a standardized evaluation framework. A concise summary of the main adhesion and mechanical assays, testing conditions, substrates, and reported parameters is provided in Table 6.

As summarized in Table 6, experimental conditions were equally heterogeneous. Most studies evaluated adhesive performance under wet physiological conditions, while others additionally examined adhesion in simulated body fluid (SBF) [25,27,31,57], blood-contact environments [24,25,35], or dry substrates [52,54,58], depending on the intended surgical application. Because substrate composition, hydration state, and testing environment substantially influence adhesive behavior, direct comparison of reported mechanical performance remains challenging. Therefore, future studies should report adhesion performance according to the intended clinical application and testing environment, distinguishing between bone defect repair, fracture fixation, implant coating, hemostatic sealing, and guided bone regeneration models.

Similarly, outcome reporting lacked uniformity. Adhesion strength was predominantly expressed in kPa, although several studies reported values in MPa, maximum force (N), or interfacial toughness (J m^−2^), while others focused primarily on rheological or hemostatic performance without providing quantitative adhesion values [47]. This inconsistency further limits direct benchmarking among different bioadhesive systems.

According to these definitions, only a limited number of studies evaluated adhesive performance under load-bearing or mechanically relevant conditions representative of clinically oriented orthopedic fixation [24,25,26,27,28,30,31,61]. Notable examples include pressure-sensitive fracture fixation systems [31], injectable polyurethane bone adhesives [27], mineral-organic bioadhesives [25], and dual-biomimetic adhesive platforms [28], all of which demonstrated promising fixation-oriented or bone-regenerative performance. Consequently, despite the substantial improvements in adhesive strength reported during the past decade, the absence of harmonized testing methodologies remains one of the principal barriers to comparing material performance and establishing clinically relevant benchmarks.

Future mechanical evaluation of bone bioadhesives should therefore move beyond isolated adhesion-strength measurements and incorporate clinically oriented testing frameworks. At minimum, studies should report standardized substrate preparation, bonded area, hydration state, curing or gelation conditions, loading mode, displacement or loading rate, failure mode, and whether failure occurred cohesively, adhesively, or within the substrate. Whenever possible, testing should be performed on bone, mineralized substrates, or validated bone-mimicking materials under wet or pseudo-physiological conditions. For fracture-related indications, adhesive systems should be evaluated in construct-level models that reproduce compression, bending, torsion, shear, cyclic loading, or displacement resistance relevant to fracture stabilization. Benchmarking against clinically used materials, including PMMA bone cement, calcium phosphate cements, commercial membranes, standard grafting materials, screws, plates, or other fixation devices, should be incorporated to determine whether new bioadhesives offer meaningful mechanical or biological advantages over current clinical options.

## 4. Translational Readiness, Limitations and Future Directions

### 4.1. Translational Readiness, Clinical Positioning and Regulatory Considerations

The translational perspective of the included literature suggests growing awareness of the requirements necessary for future clinical implementation. Most studies discuss potential clinical applications, although the reported level of translational readiness remains heterogeneous. Manufacturing feasibility, reproducibility, scalability, and practical implementation are addressed with varying levels of detail, indicating that many proposed systems remain at an early stage of development despite promising preclinical performance. Consequently, clinical positioning cannot be evaluated solely on the basis of biological or adhesive outcomes, since laboratory performance alone does not necessarily predict successful clinical adoption. Addressing these challenges requires evaluation strategies that extend beyond proof-of-concept demonstrations toward robust, clinically relevant validation.

The included studies covered a broad range of orthopedic and craniofacial applications. Counts were extracted from the final dataset of 38 included studies. Because some studies evaluated multiple models, anatomical sites, or pathological conditions, model categories were allowed to overlap. In terms of anatomical or clinical indication, craniofacial or calvarial models were the most frequent (*n* = 19), followed by long-bone or fracture-related models (*n* = 15), alveolar or extraction-socket models (*n* = 2) [25,53], mandibular models (*n* = 1) [23], segmental defect models (*n* = 2) [43,54], and other anatomical sites such as the sternum (*n* = 1) [48]. Implant-related or osseointegration models were also identified (*n* = 1) [61]. This distribution highlights the versatility of bioadhesive technologies while also indicating that current evidence remains concentrated on a limited number of experimental scenarios.

Regarding defect type and scale of in vivo validation, critical-sized defect models were frequently used, especially calvarial defects in rodents (*n* = 19). Other studies relied on non-critical defects, comminuted fractures, post-extraction sockets, or surgically created bone injuries designed primarily to assess fixation or adhesive performance rather than regenerative capacity (*n* = 16). Small-animal models predominated (*n* = 35), whereas large-animal models were scarce (*n* = 1) [24]. Small-animal models included rodents and rabbits, while the only large-animal model identified involved sheep in combination with a rat model. Studies without in vivo validation were recorded separately (*n* = 3) [28,44,50]. This distribution confirms that most evidence remains concentrated in early preclinical settings, limiting direct extrapolation to human bone repair.

With respect to biological condition, most in vivo studies evaluated healthy bone healing (*n* = 29). Disease-specific or compromised-healing models were less frequent (*n* = 6) and included diabetic bone-defect or diabetic fracture models (*n* = 3) [25,30,60], osteoporotic fracture or osteoporotic bone-defect models (*n* = 1) [34], inflammatory or infection-related bone-defect models (*n* = 1) [62], and tumor/post-resection models (*n* = 1) [52]. These categories were recorded according to the experimental condition explicitly described in each study. The narrow representation of clinically relevant pathological conditions remains an important limitation, since diabetes, osteoporosis, infection, inflammation, tumor-related bone loss, and impaired vascularization may substantially influence the performance of adhesive biomaterials.

The findings of this review also highlight the absence of standardized evaluation frameworks for bone bioadhesives, representing one of the principal challenges for interpreting and comparing published evidence. Considerable heterogeneity exists in experimental protocols, outcome measures, testing environments, and reporting practices across the included studies. This variability limits quantitative comparison between materials and complicates identification of the most promising design strategies. While individual investigations frequently report favorable results, differences in methodological approaches reduce the overall consistency of the evidence base.

### 4.2. Limitations of the Current Evidence

The evidence synthesized in this review presents several limitations that should be considered when interpreting the translational maturity of bone bioadhesive biomaterials. First, adhesion and mechanical testing protocols were highly heterogeneous across studies, with differences in substrates, hydration conditions, loading modes, bonded areas, reporting units, and failure analysis. This variability prevented quantitative pooling of adhesion values and limited direct benchmarking between materials.

Second, most in vivo studies were performed in small-animal models and non-load-bearing or low-load anatomical sites, particularly craniofacial and calvarial defects. Although these models are useful for proof-of-concept evaluation, they do not fully reproduce the mechanical, anatomical, vascular, and immunological complexity of clinically relevant human bone repair. Large-animal models, load-bearing defect models, long-term implantation studies, and disease-specific models remain limited.

Third, many systems were evaluated using early-stage biological endpoints, such as cytocompatibility, osteogenic marker expression, mineralization, or short-term histological bone formation. Fewer studies provided long-term degradation, safety, inflammatory response, sterilization, manufacturing, storage, or regulatory data. This limits the ability to determine whether promising laboratory performance can be translated into clinically deployable products.

Finally, the increasing complexity of multifunctional bioadhesive formulations may itself represent a translational barrier. Systems combining multiple polymers, nanoparticles, ions, drugs, extracellular vesicles, growth factors, or responsive chemistries may offer important biological advantages, but they also increase challenges related to reproducibility, quality control, scalability, cost, sterilization, and regulatory approval.

### 4.3. Future Directions

The trends identified throughout this review indicate that future progress will likely depend on combining advanced material engineering with standardized biological and translational evaluation. Rather than continuing to optimize isolated material properties, future developments should emphasize integrated design approaches capable of satisfying mechanical, biological, and clinical requirements simultaneously. Greater methodological harmonization would also improve comparison across studies and facilitate identification of genuinely superior technologies. Although the current evidence demonstrates substantial innovation, additional work remains necessary before the full clinical potential of multifunctional bone bioadhesives can be realized.

The next generation of bone bioadhesives will likely depend less on the incorporation of additional bioactive components and more on establishing rational design criteria that define the level of adhesion, mechanical performance, degradation kinetics, and biological activity required for specific clinical indications. Future studies should define the intended clinical indication at the design stage and select mechanical tests accordingly, distinguishing between applications such as guided bone regeneration, alveolar socket preservation, implant coating, craniofacial defect repair, hemostatic sealing, and load-relevant fracture stabilization. Rather than reporting isolated adhesion values alone, studies should benchmark adhesive performance against clinically used fixation or grafting materials and evaluate whether the adhesive provides added value in terms of fixation, handling, biological regeneration, degradation, safety, and surgical applicability. Rather than continuing the current trend of increasing compositional complexity, future research should prioritize clinically driven design, scalable manufacturing, standardized testing protocols, and robust validation in load-bearing large-animal models. These advances are expected to accelerate translation from proof-of-concept multifunctional hydrogels toward clinically deployable bone fixation technologies.

## 5. Conclusions

The studies included in this review collectively demonstrate remarkable progress in the development of bioadhesive materials for bone regeneration, with a clear transition from simple tissue adhesives toward multifunctional platforms capable of simultaneously providing wet adhesion, osteogenic stimulation, antibacterial activity, immunomodulation, and minimally invasive delivery. Hydrogel-based systems dominate the current landscape owing to their injectability, excellent tissue conformity, and ability to incorporate a wide range of bioactive components.

Despite these advances, translation remains limited by heterogeneous testing protocols, insufficient load-bearing validation, scarce large-animal and disease-specific models, limited long-term safety data, and the increasing complexity of multifunctional formulations. These limitations indicate that future development should move beyond proof-of-concept biological performance and place greater emphasis on standardized testing, clinically relevant mechanical validation, reproducible manufacturing, sterilization compatibility, regulatory feasibility, and long-term safety.

Current bioadhesives also remain largely designed according to a “one-size-fits-all” philosophy. However, bone healing is strongly influenced by patient-specific factors, including age, bone quality, metabolic status, immune response, vascularization, and defect geometry. The next generation of bone adhesives should therefore evolve toward personalized and smart biomaterials capable of adapting their mechanical properties, degradation kinetics, and therapeutic activity according to the biological environment of each patient.

Interestingly, although many studies describe their materials as mussel-inspired, most designs reproduce only a limited aspect of natural adhesion through the incorporation of catechol functional groups. Natural mussel adhesion is considerably more sophisticated, involving hierarchical protein architectures, spatial chemical gradients, sequential secretion processes, and dynamic adaptation to changing environmental conditions. More faithful biomimetic strategies that capture these multiscale mechanisms may provide opportunities to further improve adhesive performance under challenging physiological conditions.

Overall, the current evidence indicates that bone bioadhesives have evolved into highly multifunctional regenerative platforms with considerable clinical potential. Nevertheless, successful translation into routine orthopedic, craniofacial, and dental practice will require not only improved biological performance, but also long-term mechanical reliability under physiologically relevant conditions, simplified and reproducible formulations, standardized regulatory evaluation pathways, and scalable manufacturing processes. Addressing these challenges will be essential for transforming bone bioadhesives from promising experimental materials into clinically viable technologies for next-generation bone repair and regeneration.

## Figures and Tables

**Table 1:** Main factors limiting clinical translation of bone-specific bioadhesive biomaterials.

Translational Factor	Relevance for Clinical Translation	Examples of Current Limitations	Supporting References
**Bone-specific wet adhesion**	Adhesives must bond to hydrated, mineralized, porous, and biologically active bone surfaces.	Adhesion often tested on non-bone substrates, dry surfaces, or simplified tissue models.	[13,19,20,21,24,25]
**Mechanical relevance and load-bearing capacity**	Bone adhesives should resist displacement under fixation, defect repair, implant, or fracture-related loading.	Limited evaluation under bending, torsion, cyclic loading, or construct-level fixation conditions.	[24,25,27,28,30,31]
**Standardization of adhesion testing**	Comparable metrics are needed to identify superior material strategies.	Heterogeneous lap-shear, tensile, peel, burst-pressure, push-out, and custom-made assays.	[13,20,21,24,25]
**Benchmarking against clinical materials**	New adhesives should be compared with current fixation or grafting options.	Limited comparison with PMMA, calcium phosphate cements, commercial membranes, grafts, plates, or screws.	[10,11,12,24,25,27]
**Biological multifunctionality**	Clinical success requires not only adhesion but also osteogenesis, angiogenesis, immunomodulation, antibacterial activity, and controlled degradation.	Some studies emphasize adhesive strength but provide limited biological or long-term regenerative validation.	[22,26,28,32,33,34,35,36]
**Preclinical model relevance**	Models should reflect clinically relevant anatomy, defect size, loading, and pathological conditions.	Predominance of small-animal, non-load-bearing, healthy bone-defect models.	[5,7,8,9,13,25,32,35,36,37]
**Long-term safety and degradation**	Adhesives should degrade predictably and avoid chronic inflammation, toxicity, or unstable interfaces.	Limited long-term implantation, degradation, and safety data.	[5,7,13,20,21]
**Manufacturing, sterilization, and scalability**	Translation requires reproducible fabrication, storage stability, sterilization compatibility, and regulatory feasibility.	Complex multifunctional formulations may increase production, quality-control, and regulatory burden.	[7,8,9,13,17,20]

**Table 2:** Data extraction variables used in the systematic review.

Domain	Extracted Variables
**Study characteristics**	Author, year, journal, model, defect type, comparator
**Material design**	Platform, composition, bioinspired motif, nanocomposite, cargo
**Adhesion/mechanics**	Adhesion mechanism, assay, substrate, condition, strength value
**Biological function**	Osteogenesis, angiogenesis, immunomodulation, antibacterial activity
**In vivo validation**	Species, defect, follow-up, bone formation, safety
**Translation**	Load-bearing testing, scalability, regulation, limitations

**Table 3:** Polymeric matrices identified across the included bioadhesive systems.

Category	Polymer/Subcategory	Included Studies
**Natural polymers**	Gelatin	[31,33,34,35,36,40,41,42,43,44]
	Chitosan	[26,45,46,47,48,49,50]
	Silk fibroin	[24,32,37,51]
	Alginate	[25,32,52]
	Collagen	[22]
	Hyaluronic acid	[23,33]
	Dextran-containing systems	[28,35,40,42]
**Synthetic polymers**	PEG	[32,53,54,55,56,57]
	Polyurethane	[27,58]
	OPF	[55]
	Poly(NAGA-co-AANHS)	[59]
**Bioinspired synthetic polymer**	Polydopamine	[30]

**Table 4:** Structural reinforcement strategies.

Structural Reinforcement	Representative Reinforcement Phase	Studies
**Nanoclay**	Laponite (native or functionalized)	[22,32,34,47]
**Bioactive glass**	Mesoporous bioactive glass nanoparticles	[33,42,57]
**Hydroxyapatite-based nanoparticles**	Hydroxyapatite, nano-HAp, doped HAp	[24,27,28,31,40,48,58,60]
**Silica nanoparticles**	Mesoporous silica nanoparticles	[41]
**MXene nanosheets**	Ti_3_C_2_ MXene	[35]
**Black phosphorus nanosheets**	BP@TA-Mg	[37]
**Graphene oxide**	GO nanosheets	[49]
**ZIF nanoparticles**	ZIF-8	[26]
**Layered double hydroxides**	PDA-coated LDH	[56]
**Polymer fibers**	PDA-coated HAp/PLLA fibers	[43]
**Bredigite nanoparticles**	Bredigite	[30]
**Copper oxide nanoparticles**	CuO	[57]
**Silver nanoparticles**	TA@Ag nanoparticles	[32]
**Amorphous calcium phosphate**	ACP nanoparticles	[25,51]

**Table 5:** Bioactive cargo incorporated into bioadhesive systems.

Bioactive Cargo	Representative Agents	Studies
**Growth factors**	BMP-2	[54,60]
**Extracellular vesicles**	Osteogenic EVs	[23]
**MSC-derived nanovesicles**	Melatonin-loaded nMSC vesicles	[53]
**Cell-derived extracts**	Cell-free fat extract	[43]
**Anticancer drugs**	Doxorubicin	[50]
**Antiresorptive drugs**	Alendronate	[41]
**Flavonoids**	Dihydromyricetin; Rutin	[44,45]
**Bioactive peptide**	Cod peptides	[40]
**Metabolic molecules**	β-Hydroxybutyrate	[59]
**Therapeutic ions**	Ca^2+^, Sr^2+^, Mg^2+^, PO_4_^3−^, Ag^+^	[46,52]

**Table 6:** Summary of adhesion and mechanical testing approaches identified across the included studies.

Assay/Method	Typical Testing Condition	Common Substrates/Constructs	Main Reported Parameter	Main Limitation for Comparison
**Lap-shear adhesion**	Dry, wet, SBF, or blood-contact conditions	Bone, skin, glass, titanium, PMMA, mineralized or polymeric substrates	Adhesion strength, usually kPa or MPa	Highly dependent on bonded area, substrate preparation, hydration state, and loading rate
**Tensile adhesion**	Wet or pseudo-physiological conditions	Tissue/material interfaces, bone-like substrates, hydrogels, coatings	Tensile adhesion strength or maximum force	Sensitive to alignment, gripping, sample geometry, and failure mode
**Peel testing**	Wet or hydrated conditions	Flexible membranes, coatings, hydrogel films, tissue-like substrates	Peel force or work of adhesion	Mainly applicable to flexible interfaces; limited comparability with rigid bone fixation
**Push-out/pull-out testing**	Bone-like or implant-related conditions	Bone–implant interfaces, coatings, scaffolds, defect constructs	Push-out force, pull-out force, fixation strength	Reflects construct-level fixation rather than purely interfacial adhesion
**Burst pressure/sealing tests**	Wet or fluid-pressure conditions	Membranes, sealants, tissue defects, hemostatic interfaces	Burst pressure or sealing pressure	More relevant to sealing/hemostasis than load-bearing bone fixation
**Compression/bending/torsion**	Dry, wet, or bone-relevant construct conditions	Hydrogels, scaffolds, fracture models, adhesive–bone constructs	Compressive strength, modulus, bending strength, construct stability	Measures bulk or construct mechanics, not necessarily interfacial adhesion
**Rheology/injectability**	Pre-gel, gelation, or hydrated conditions	Injectable hydrogels and precursor solutions	Storage modulus, loss modulus, gelation time, viscosity	Describes handling and network formation but not direct bone adhesion
**Interfacial toughness tests**	Hydrated or fracture-mechanics-based conditions	Bonded interfaces, hydrogel–substrate systems, mineralized substrates	Work of adhesion or interfacial toughness, J m^−2^	Strongly dependent on crack geometry, loading mode, and fracture path

## Data Availability

The original contributions presented in this study are included in the article/Supplementary Material. Further inquiries can be directed to the corresponding authors.

## Supplementary Materials

The following supporting information can be downloaded at: https://www.mdpi.com/article/10.3390/polym18182266/s1, Supplementary File S1. Database-specific search equations. Supplementary File S2. Supplementary sensitivity search terms. Table S1. Developmental classification of the 38 included studies according to functional and translational complexity. Table S2. Predominant bioadhesion mechanisms identified in the included studies. Table S3. Full-text eligibility assessment of included and excluded studies with reasons for inclusion or exclusion. Supplementary File S3. PRISMA 2020 checklist.
