# Supplementary material for: Natural and Hybrid Bioadhesive Polymeric Platforms for Bone Regeneration: A PRISMA-Guided Systematic Review of Design Strategies and Translational Challenges
Source: Polymers (Basel). 2026 Sep 17;18(18):2266. doi: 10.3390/polym18182266 (PMC13611712; doi:10.3390/polym18182266)
Supplement: Supplementary file 1 [file polymers-18-02266-s001.zip › Supplementary File S1-S2.pdf]

Supplementary Material

Supplementary File S1. Database-specific search equations

The final search was performed on 11 May 2026.

S1. PubMed/MEDLINE

((bioadhesive*[Title/Abstract] OR "tissue adhesive"[Title/Abstract] OR "bone adhesive"[Title/Abstract] OR "adhesive hydrogel"[Title/Abstract] OR "wet adhesive"[Title/Abstract] OR "tissue sealant"[Title/Abstract] OR "surgical sealant"[Title/Abstract] OR catechol[Title/Abstract] OR "mussel-inspired"[Title/Abstract] OR "injectable adhesive"[Title/Abstract] OR "self-healing hydrogel"[Title/Abstract] OR "adhesive scaffold"[Title/Abstract]) AND (smart[Title/Abstract] OR multifunctional[Title/Abstract] OR "stimuli-responsive"[Title/Abstract] OR responsive[Title/Abstract] OR immunomodulatory[Title/Abstract] OR bioactive[Title/Abstract] OR osteoimmunomodulatory[Title/Abstract] OR "controlled release"[Title/Abstract] OR antibacterial[Title/Abstract] OR angiogenic[Title/Abstract]) AND (hydrogel*[Title/Abstract] OR scaffold*[Title/Abstract] OR nanocomposite*[Title/Abstract] OR composite*[Title/Abstract] OR "hybrid composite"[Title/Abstract] OR biomaterial*[Title/Abstract]) AND ("bone regeneration"[Title/Abstract] OR "bone tissue engineering"[Title/Abstract] OR bone defect*[Title/Abstract] OR "guided bone regeneration"[Title/Abstract] OR craniofacial bone[Title/Abstract] OR mandibular bone[Title/Abstract] OR "alveolar bone regeneration"[Title/Abstract] OR osteogenesis[Title/Abstract] OR osteogenic[Title/Abstract] OR "bone repair"[Title/Abstract] OR "bone healing"[Title/Abstract] OR fracture*[Title/Abstract] OR "fracture repair"[Title/Abstract] OR osseointegration[Title/Abstract]) AND (adhesion[Title/Abstract] OR "adhesive strength"[Title/Abstract] OR "interfacial bonding"[Title/Abstract] OR "cohesive strength"[Title/Abstract] OR crosslink*[Title/Abstract]) NOT (review[Publication Type] OR editorial[Publication Type] OR letter[Publication Type]) NOT (cartilage[Title/Abstract] OR chondro*[Title/Abstract] OR prosthe*[Title/Abstract] OR orthopedic*[Title/Abstract] OR orthopaedic*[Title/Abstract]) NOT (periodontitis[Title/Abstract] OR "periodontal disease"[Title/Abstract]) AND ("2016/01/01"[Date - Publication] : "2026/05/11"[Date - Publication]) AND english[Language]

S2. Web of Science

TS=(
bioadhesive* OR "tissue adhesive" OR "bone adhesive" OR "adhesive hydrogel" OR "wet adhesive" OR "tissue sealant" OR "surgical sealant" OR catechol OR "mussel-inspired" OR "injectable adhesive" OR "self-healing hydrogel" OR "adhesive scaffold"
)
AND TS=(
smart OR multifunctional OR "stimuli-responsive" OR responsive OR immunomodulatory OR bioactive OR osteoimmunomodulatory OR "controlled release" OR antibacterial OR angiogenic
)
AND TS=(
hydrogel* OR scaffold* OR nanocomposite* OR composite* OR "hybrid composite" OR biomaterial*
)
AND TS=(
"bone regeneration" OR "bone tissue engineering" OR "bone defect*" OR "guided bone regeneration" OR craniofacial bone OR mandibular bone OR "alveolar bone regeneration" OR osteogenesis OR osteogenic OR "bone repair" OR "bone healing" OR fracture* OR "fracture repair" OR osseointegration
)
AND TS=(
adhesion OR "adhesive strength" OR "interfacial bonding" OR "cohesive strength" OR crosslink*
)
NOT TS=(
cartilage OR chondro* OR prosthe* OR orthopedic* OR orthopaedic* OR periodontitis OR "periodontal disease"
)
AND PY=(2016-2026)
AND LA=(English)
AND DT=(Article)

S3. Scopus

TITLE-ABS-KEY(
(
bioadhesive* OR "tissue adhesive" OR "bone adhesive" OR "adhesive hydrogel" OR "wet adhesive" OR "tissue sealant" OR "surgical sealant" OR catechol OR "mussel-inspired" OR "injectable adhesive" OR "self-healing hydrogel" OR "adhesive scaffold"
)
AND
(
smart OR multifunctional OR "stimuli-responsive" OR responsive OR immunomodulatory OR bioactive OR osteoimmunomodulatory OR "controlled release" OR antibacterial OR angiogenic
)
AND
(
hydrogel* OR scaffold* OR nanocomposite* OR composite* OR "hybrid composite" OR biomaterial*
)
AND
(
"bone regeneration" OR "bone tissue engineering" OR "bone defect*" OR "guided bone regeneration" OR craniofacial bone OR mandibular bone OR "alveolar bone regeneration" OR osteogenesis OR osteogenic OR "bone repair" OR "bone healing" OR fracture* OR "fracture repair" OR osseointegration
)
AND
(
adhesion OR "adhesive strength" OR "interfacial bonding" OR "cohesive strength" OR crosslink*
)
)
AND NOT TITLE-ABS-KEY(
cartilage OR chondro* OR prosthe* OR orthopedic* OR orthopaedic* OR periodontitis OR "periodontal disease"
)
AND PUBYEAR > 2015 AND PUBYEAR < 2027
AND LIMIT-TO(LANGUAGE, "English")
AND LIMIT-TO(DOCTYPE, "ar")

S4. IEEE Xplore

("All Metadata":(
bioadhesive OR "tissue adhesive" OR "bone adhesive" OR "adhesive hydrogel" OR "wet adhesive" OR "tissue sealant" OR "surgical sealant" OR catechol OR "mussel-inspired" OR "injectable adhesive" OR "self-healing hydrogel" OR "adhesive scaffold"
))
AND
("All Metadata":(
smart OR multifunctional OR "stimuli-responsive" OR responsive OR immunomodulatory OR bioactive OR osteoimmunomodulatory OR "controlled release" OR antibacterial OR angiogenic
))
AND
("All Metadata":(
hydrogel OR scaffold OR nanocomposite OR composite OR "hybrid composite" OR biomaterial
))
AND
("All Metadata":(
"bone regeneration" OR "bone tissue engineering" OR "bone defect*" OR "guided bone regeneration" OR craniofacial bone OR mandibular bone OR "alveolar bone regeneration" OR osteogenesis OR osteogenic OR "bone repair" OR "bone healing" OR fracture* OR "fracture repair" OR osseointegration
))
AND
("All Metadata":(

adhesion OR "adhesive strength" OR "interfacial bonding" OR "cohesive strength" OR crosslink
))
NOT
("All Metadata":(
cartilage OR chondro* OR prosthe* OR orthopedic* OR orthopaedic* OR periodontitis OR "periodontal disease"
))
AND ("Publication Year":2016-2026)

Supplementary File S2. Supplementary sensitivity search terms

A supplementary sensitivity search was conducted using the following additional terms: “bone glue”, “osteoadhesive”, “fracture adhesive”, “mineral-organic adhesive”, “adhesive periosteum”, “adhesive membrane”, and “interfacial toughness”. These terms were searched in PubMed/MEDLINE, Scopus, Web of Science, and IEEE Xplore within the same date range as the primary search. Retrieved records were checked against the final included dataset and assessed according to the same eligibility criteria.
This supplementary sensitivity search did not identify additional eligible studies beyond those already included in the final qualitative synthesis.

Table S1. Developmental classification of the 38 included studies according to functional and translational complexity.

Reference	Proposed developmental category	Classification rationale
Cheng et al., 2025	**Type III—Multifunctional regenerative platform**	Adhesion + osteoimmunomodulation + osteogenesis.
Chen et al., 2025	**Type IV—Smart multifunctional platform**	ROS-responsive + osteogenesis + antibacterial + immunomodulation.
Zhao et al., 2026	**Type IV—Smart multifunctional platform**	Metabolic-responsive + osteogenesis + osteoimmune regulation.
Yu et al., 2026	**Type III—Multifunctional regenerative platform**	Adhesion + osteogenesis + angiogenesis + immunomodulation.
Li et al., 2026	**Type III—Multifunctional regenerative platform**	Adhesion + osteogenesis + antibacterial activity.
Liang et al., 2026	**Type III—Multifunctional regenerative platform**	Adhesion + osteogenesis + angiogenesis + osteoimmunomodulation.
Lei et al., 2026	**Type III—Multifunctional regenerative platform**	Adhesion + osteogenesis + angiogenesis + immunomodulation.
Wu et al., 2023	**Type III—Multifunctional regenerative platform**	Adhesion + osteogenesis + osteoimmune modulation.
Yan et al., 2026	**Type IV—Smart multifunctional platform**	NIR-responsive + osteogenesis + angiogenesis + antibacterial activity.
Yang et al., 2023	**Type III—Multifunctional regenerative platform**	Adhesion + osteogenesis + antibacterial activity.
Liu et al., 2019	**Type III—Multifunctional regenerative platform**	Adhesion + osteogenesis + antibacterial activity + drug delivery.
Tong et al., 2026	**Type II—Bioactive/regenerative adhesive**	Adhesion + osteogenesis only.
Hu et al., 2026	**Type III—Multifunctional regenerative platform**	Adhesion + osteogenesis + angiogenesis + antibacterial activity.
Yuan et al., 2025	**Type II—Bioactive/regenerative adhesive**	Adhesion + hemostatic/bioactive function without multifunctionality.
Zhang et al., 2021	**Type II—Bioactive/regenerative adhesive**	Adhesion + osteogenesis only.
Hou et al., 2022	**Type II—Bioactive/regenerative adhesive**	Adhesion + osteogenesis only.
Bai et al., 2020	**Type II—Bioactive/regenerative adhesive**	Adhesion + osteogenesis only.
Ma et al., 2023	**Type II—Bioactive/regenerative adhesive**	Adhesion + osteogenesis only.
Yang et al., 2024	**Type III—Multifunctional regenerative platform**	Adhesion + osteogenesis + antioxidant activity.
Shokri et al., 2024	**Type III—Multifunctional regenerative platform**	Adhesion + osteogenesis + antibacterial + hemostatic activity.
Huang et al., 2023	**Type IV—Smart multifunctional platform**	NIR-responsive + osteogenesis + immunomodulation.
Shi et al., 2025	**Type II—Bioactive/regenerative adhesive**	Adhesion + osteogenesis only.
Xing et al., 2025	**Type IV—Smart multifunctional platform**	ROS-responsive + osteogenesis + anti-inflammatory activity.
Yang et al., 2025	**Type II—Bioactive/regenerative adhesive**	Adhesion + osteogenesis only.
Moazami et al., 2023	**Type III—Multifunctional regenerative platform**	Adhesion + osteogenesis + antibacterial activity.
Wang et al., 2021	**Type III—Multifunctional regenerative platform**	Adhesion + osteogenesis + self-healing functionality.
Li et al., 2026	**Type II—Bioactive/regenerative adhesive**	Adhesion + osseointegration/osteogenesis only.
Wang et al., 2024	**Type III—Multifunctional regenerative platform**	Adhesion + osteogenesis + immunomodulation.
Yang et al., 2022	**Type III—Multifunctional regenerative platform**	Adhesion + osteogenesis + angiogenesis.

Duan et al., 2025	**Type IV—Smart multifunctional platform**	Enzyme-responsive + osteogenesis + antibacterial activity.
Yun et al., 2025	**Type IV—Smart multifunctional platform**	Light-responsive + osteogenesis.
Liu et al., 2020	**Type III—Multifunctional regenerative platform**	Adhesion + osteogenesis + angiogenesis + antibacterial activity.
Zhang et al., 2025	**Type IV—Smart multifunctional platform**	pH-responsive + antitumor + osteogenic activity.
George et al., 2022	**Type IV—Smart multifunctional platform**	pH-responsive + osteogenesis.
Che et al., 2026	**Type III—Multifunctional regenerative platform**	Adhesion + osteogenesis + angiogenesis + hemostatic activity.
Chen et al., 2025	**Type IV—Smart multifunctional platform**	ROS-responsive + osteogenesis + antibacterial + immunomodulation.
Huang et al., 2016	**Type II—Bioactive/regenerative adhesive**	Adhesion + osteogenesis only.
Cespedes-Valenzuela et al., 2022	**Type IV—Smart multifunctional platform**	Light-responsive + osteogenesis + antibacterial activity.

Table S2. Predominant bioadhesion mechanisms identified in the included studies.

Reference	Predominant bioadhesion mechanism(s)
Cheng et al., 2025	Covalent bonding
Chen et al., 2025	Supramolecular interactions
Zhao et al., 2026	Covalent bonding
Yu et al., 2026	Covalent bonding
Li et al., 2026	Covalent bonding
Liang et al., 2026	Covalent bonding
Lei et al., 2026	Hydrogen bonding; Supramolecular interactions
Wu et al., 2023	Covalent bonding; Catechol chemistry
Yan et al., 2026	Covalent bonding
Yang et al., 2023	Covalent bonding
Liu et al., 2019	Electrostatic interactions
Tong et al., 2026	Covalent bonding
Hu et al., 2026	Covalent bonding
Yuan et al., 2025	Hydrogen bonding
Zhang et al., 2021	Hydrogen bonding
Hou et al., 2022	Covalent bonding
Bai et al., 2020	Hydrogen bonding
Ma et al., 2023	Covalent bonding
Yang et al., 2024	Hydrogen bonding
Shokri et al., 2024	Metal coordination; Catechol chemistry
Huang et al., 2023	Not explicitly identified
Shi et al., 2025	Hydrogen bonding
Xing et al., 2025	Covalent bonding
Yang et al., 2025	Covalent bonding
Moazami et al., 2023	Metal coordination; Catechol chemistry
Wang et al., 2021	Covalent bonding
Li et al., 2026	Catechol chemistry
Wang et al., 2024	Not explicitly identified
Yang et al., 2022	Hydrogen bonding; Supramolecular interactions
Duan et al., 2025	Covalent bonding
Yun et al., 2025	Catechol chemistry
Liu et al., 2020	Covalent bonding; Catechol chemistry
Zhang et al., 2025	Covalent bonding; Hydrogen bonding
George et al., 2022	Hydrogen bonding
Che et al., 2026	Not explicitly identified
Chen et al., 2025	Not explicitly identified
Huang et al., 2016	Covalent bonding
Cespedes-Valenzuela et al., 2022	Hydrogen bonding; Covalent bonding; Electrostatic interactions; Mechanical interlocking

Table S3. Full-text eligibility assessment of included and excluded studies with reasons for inclusion or exclusion.

No.	First author	Year	Short title / study identifier	Reference (DOI)	Final decision	Reason for inclusion or exclusion
ART-001	Xuefeng Zhou	2020	Ca ions chelation, collagen I incorporation and 3D bionic PLGA/PCL electrospun architecture to enhance osteogenic differentiation	10.1016/j.matdes.2020.109300	Exclude	Studies evaluating conventional bone cements, fixation devices, implant coatings, or scaffolds without a primary adhesive function or adhesion-related evaluation.
ART-002	Junyao Cheng	2025	Dual-Biomimetic Bone Adhesive with Osteoimmunomodulatory Capabilities for Anatomical Reconstruction of Comminuted Fractures	10.1002/advs.202501108	Include	Bioadhesive/interfacial biomaterial with direct bone-regeneration relevance and experimental validation.
ART-003	Kaixuan Ma	2024	Mussel-inspired multi-bioactive microsphere scaffolds for bone defect photothermal therapy	10.1016/j.mtbio.2024.101363	Exclude	Studies not reporting any evaluation of adhesive performance when adhesion is a claimed functional property.
ART-004	Xu Chen	2025	Nano-assisted dynamically assembled hydrogels with strong tissue adhesion and proactive immunomodulation for bone defect repair	10.1016/j.bioactmat.2025.07.038	Include	Bioadhesive/interfacial biomaterial with direct bone-regeneration relevance and experimental validation.
ART-005	Jiaxin Li	2023	Self-healing hybrid hydrogels with sustained bioactive components release for guided bone regeneration	10.1186/s12951-023-01811-8	Exclude	Studies not reporting any evaluation of adhesive performance when adhesion is a claimed functional property.
ART-006	Zifan Zhao	2026	Wet-adhesive metabolic hydrogel for osteoimmune-guided extraction socket healing	10.1016/j.mtbio.2026.102943	Include	Bioadhesive/interfacial biomaterial with direct bone-regeneration relevance and experimental validation.
ART-007	Tingting Yu	2026	MSC-mimicking nanovesicle embedded bio-adhesive hydrogel for dual immunomodulation and osteogenesis to promote maxillofacial bone regeneration	10.1016/j.bioactmat.2026.02.032	Include	Bioadhesive/interfacial biomaterial with direct bone-regeneration relevance and experimental validation.
ART-008	Shanshan Li	2026	Multifunctional injectable chitosan-based adhesive enhanced by limited oxidation for assisting bone grafting	10.1016/j.mtbio.2026.102759	Include	Bioadhesive/interfacial biomaterial with direct bone-regeneration relevance and experimental validation.
ART-009	Zhaoquan Liang	2026	Mussel-inspired adhesive hydrogel reprograms osteoimmune niche for robust bone regeneration in complex defects	10.1016/j.mtbio.2026.102996	Include	Bioadhesive/interfacial biomaterial with direct bone-regeneration relevance and experimental validation.
ART-010	Puzhou Lei	2025	Pressure-sensitive composite bandages with high toughness, self-healability and strong tissues adhesion for rapid bone fixation and accelerated osteogenesis via immunomodulation and neovascularization	10.1016/j.mtbio.2025.102730	Include	Bioadhesive/interfacial biomaterial with direct bone-regeneration relevance and experimental validation.
ART-011	Minhao Wu	2023	Engineering mussel-inspired multifunctional nanocomposite hydrogels to orchestrate osteoimmune microenvironment and promote bone healing	10.1016/j.matdes.2023.111705	Include	Bioadhesive/interfacial biomaterial with direct bone-regeneration relevance and experimental validation.
ART-012	Yue Yan	2025	Advanced injectable Ti_3C_2 MXene hydrogel: A multifunctional solution for diabetic alveolar bone defect repair	10.1016/j.cej.2025.171850	Include	Bioadhesive/interfacial biomaterial with direct bone-regeneration relevance and experimental validation.
ART-013	Yusheng Yang	2023	Triple-functional bone adhesive with enhanced internal fixation, bacteriostasis and osteoinductive properties for open fracture repair	10.1016/j.bioactmat.2023.01.021	Include	Bioadhesive/interfacial biomaterial with direct bone-regeneration relevance and experimental validation.
ART-014	Lili Liu	2019	Adhesive liposomes loaded onto an injectable, self-healing and antibacterial hydrogel for promoting bone reconstruction	10.1038/s41427-019-0185-z	Include	Bioadhesive/interfacial biomaterial with direct bone-regeneration relevance and experimental validation.
ART-015	Lei Tong	2026	Constructing highly bioactive and tough polyurethane bone adhesives by covalently integrating nanohydroxyapatite	10.1016/j.biomaterials.2026.124127	Include	Bioadhesive/interfacial biomaterial with direct bone-regeneration relevance and experimental validation.
ART-016	Wenhao Hu	2025	Robust adhesive, antibacterial, pro-angiogenic and osteogenic bone adhesives with moderate degradability facilitating bone regeneration	10.1016/j.bioactmat.2025.08.031	Include	Bioadhesive/interfacial biomaterial with direct bone-regeneration relevance and experimental validation.
ART-017	Jingjing Yuan	2025	A multifunctional highly adhesive hydrogel mimicking snail mucus for hemostatic coating	10.1016/j.cej.2025.160110	Include	Bioadhesive/interfacial biomaterial with direct bone-regeneration relevance and experimental validation.
ART-018	Hongjie Zhang	2020	A simple hydrogel scaffold with injectability, adhesivity and osteogenic activity for bone regeneration	10.1039/d0bm01840f	Include	Bioadhesive/interfacial biomaterial with direct bone-regeneration relevance and experimental validation.
ART-019	Fushan Hou	2021	Biodegradable dual-crosslinked adhesive glue for fixation and promotion of osteogenesis	10.1016/j.cej.2021.132000	Include	Bioadhesive/interfacial biomaterial with direct bone-regeneration relevance and experimental validation.
ART-020	Shumeng Bai	2020	Bioinspired Mineral-Organic Bone Adhesives for Stable Fracture Fixation and Accelerated Bone Regeneration	10.1002/adfm.201908381	Include	Bioadhesive/interfacial biomaterial with direct bone-regeneration relevance and experimental validation.
ART-021	Wuchao Ma	2023	Bioinspired self-healing injectable nanocomposite hydrogels based on oxidized dextran and gelatin for growth-factor-free bone regeneration	10.1016/j.ijbiomac.2023.126145	Include	Bioadhesive/interfacial biomaterial with direct bone-regeneration relevance and experimental validation.
ART-022	Jiali Yang	2024	Dihydromyricetin-loaded oxidized polysaccharide/L-arginine chitosan adhesive hydrogel promotes bone regeneration by regulating PI3K/AKT signaling pathway and MAPK signaling pathway	10.1016/j.carbpol.2024.122614	Include	Bioadhesive/interfacial biomaterial with direct bone-regeneration relevance and experimental validation.
ART-023	Mahshid Shokri	2023	Engineering Wet-Resistant and Osteogenic Nanocomposite Adhesive to Control Bleeding and Infection after Median Sternotomy	10.1002/adhm.202304349	Include	Bioadhesive/interfacial biomaterial with direct bone-regeneration relevance and experimental validation.

ART-024	Jinfeng Huang	2023	Harnessing immunomodulation for efficient bone Regeneration: Bioengineered black phosphorus-incorporated Self-Healing hydrogel	10.1016/j.cej.2023.144117	Include	Bioadhesive/interfacial biomaterial with direct bone-regeneration relevance and experimental validation.
ART-025	Shijie Shi	2025	In situ fluid-to-solid converting injectable wet adhesive for comminuted fracture repair via water-activated bonding	10.1016/j.actbio.2025.09.018	Include	Bioadhesive/interfacial biomaterial with direct bone-regeneration relevance and experimental validation.
ART-026	Xudan Xing	2024	Injectable bioresponsive bone adhesive hydrogels inhibit NLRP3 inflammasome on demand to accelerate diabetic fracture healing	10.1016/j.biomaterials.2024.123059	Include	Bioadhesive/interfacial biomaterial with direct bone-regeneration relevance and experimental validation.
ART-027	Lin Yang	2025	Injectable Bioactive Polyurethane Adhesive for Critical-Size Cranial Defect Restoration	10.1021/acs.chemmater.5c01618	Include	Bioadhesive/interfacial biomaterial with direct bone-regeneration relevance and experimental validation.
ART-028	Shima Moazami	2022	Multifunctional Bioinspired Bredigite-Modified Adhesive for Bone Fracture Healing	10.1021/acsami.2c20038	Include	Bioadhesive/interfacial biomaterial with direct bone-regeneration relevance and experimental validation.
ART-029	Bo Wang	2021	Mussel-Inspired Bisphosphonated Injectable Nanocomposite Hydrogels with Adhesive, Self-Healing, and Osteogenic Properties for Bone Regeneration	10.1021/acsami.1c06058	Include	Bioadhesive/interfacial biomaterial with direct bone-regeneration relevance and experimental validation.
ART-030	Jing Li	2025	Mussel-inspired silica, strontium, and fluoride-doped hydroxyapatite/BMP-2 composite coating: a new strategy to enhance the bioactivity of zirconia implants	10.1039/d5ra09896c	Include	Bioadhesive/interfacial biomaterial with direct bone-regeneration relevance and experimental validation.
ART-031	Li Wang	2023	Osteoinductive Dental Pulp Stem Cell-Derived Extracellular Vesicle-Loaded Multifunctional Hydrogel for Bone Regeneration	10.1021/acsnano.3c11542	Include	Bioadhesive/interfacial biomaterial with direct bone-regeneration relevance and experimental validation.
ART-032	Zhen Yang	2022	Self-Adhesive Hydrogel Biomimetic Periosteum to Promote Critical-Size Bone Defect Repair via Synergistic Osteogenesis and Angiogenesis	10.1021/acsami.2c08400	Include	Bioadhesive/interfacial biomaterial with direct bone-regeneration relevance and experimental validation.
ART-033	Dongming Duan	2025	Self-sacrificial bioenzyme-reinforced injectable hydrogel bone adhesives for enhancing diabetic fracture healing	10.1016/j.cej.2025.160255	Include	Bioadhesive/interfacial biomaterial with direct bone-regeneration relevance and experimental validation.
ART-034	Jinyoung Yun	2024	Visible light-induced simultaneous bioactive amorphous calcium phosphate mineralization and in situ crosslinking of coacervate-based injectable underwater adhesive hydrogels for enhanced bone regeneration	10.1016/j.biomaterials.2024.122948	Include	Bioadhesive/interfacial biomaterial with direct bone-regeneration relevance and experimental validation.
ART-035	Yanhua Liu	2020	ZIF-8-Modified Multifunctional Bone-Adhesive Hydrogels Promoting Angiogenesis and Osteogenesis for Bone Regeneration	10.1021/acsami.0c12090	Include	Bioadhesive/interfacial biomaterial with direct bone-regeneration relevance and experimental validation.
ART-036	Nahal Abie	2024	Designing of a Multifunctional 3D-Printed Biomimetic Theragenerative Aerogel Scaffold via Mussel-Inspired Chemistry: Bioactive Glass Nanofiber-Incorporated Self-Assembled Silk Fibroin with Antibacterial, Antiosteosarcoma, and Osteoinductive Properties	10.1021/acsami.4c00065	Exclude	Studies evaluating conventional bone cements, fixation devices, implant coatings, or scaffolds without a primary adhesive function or adhesion-related evaluation.
ART-037	Shuoshuo Yang	2022	Mussel inspired multifunctional bovine serum albumin (BSA) coatings loaded with Baicalein (BAI) to enhance osteogenesis and resist oxidative stress for potential application on implant	10.1016/j.ijbiomac.2022.12.285	Exclude	Studies evaluating conventional bone cements, fixation devices, implant coatings, or scaffolds without a primary adhesive function or adhesion-related evaluation.
ART-038	Xiang Li	2019	Polydopamine-Assisted Anchor of Chitosan onto Porous Composite Scaffolds for Accelerating Bone Regeneration	10.1021/acsbiomaterials.9b00209	Exclude	Studies evaluating conventional bone cements, fixation devices, implant coatings, or scaffolds without a primary adhesive function or adhesion-related evaluation.
ART-039	Xiang Gao	2016	Bioinspired Design of Polycaprolactone Composite Nanofibers as Artificial Bone Extracellular Matrix for Bone Regeneration Application	10.1021/acsami.6b10417	Exclude	Studies evaluating conventional bone cements, fixation devices, implant coatings, or scaffolds without a primary adhesive function or adhesion-related evaluation.
ART-040	Qiang Zhang	2025	Dual-functional injectable adhesive hydrogel delivering ginger-derived doxorubicin vesicles for osteosarcoma recurrence suppression and post-resection wound healing	10.3389/fbioe.2025.1609673	Include	Bioadhesive/interfacial biomaterial with direct bone-regeneration relevance and experimental validation.
ART-041	Xin Xing	2021	Mussel-inspired functionalization of electrospun scaffolds with polydopamine-assisted immobilization of mesenchymal stem cells-derived small extracellular vesicles for enhanced bone regeneration	10.1016/j.ijpharm.2021.121136	Exclude	Studies evaluating conventional bone cements, fixation devices, implant coatings, or scaffolds without a primary adhesive function or adhesion-related evaluation.
ART-042	Hyun-Ji Park	2016	Catechol-Functionalized Hyaluronic Acid Hydrogels Enhance Angiogenesis and Osteogenesis of Human Adipose-Derived Stem Cells in Critical Tissue Defects	10.1021/acs.biomac.5b01670	Exclude	Studies evaluating conventional bone cements, fixation devices, implant coatings, or scaffolds without a primary adhesive function or adhesion-related evaluation.
ART-043	Zahid Hussain	2025	Polydopamine-assisted biomineralized fibrous membrane and short fiber-reinforced spheroids for bone bioengineering	10.1016/j.bioadv.2025.214568	Exclude	Studies evaluating conventional bone cements, fixation devices, implant coatings, or scaffolds without a primary adhesive function or adhesion-related evaluation.
ART-044	Yanyi Xu	2017	Modification of Poly(propylene fumarate)-Bioglass Composites with Peptide Conjugates to Enhance Bioactivity	10.1021/acs.biomac.7b00828	Exclude	Studies evaluating conventional bone cements, fixation devices, implant coatings, or scaffolds without a primary adhesive function or adhesion-related evaluation.

ART-045	Matthew N George	2021	Injectable pH-responsive adhesive hydrogels for bone tissue engineering inspired by the underwater attachment strategy of marine mussels	10.1016/j.msec.2021.112606	Include	Bioadhesive/interfacial biomaterial with direct bone-regeneration relevance and experimental validation.
ART-046	Lingbin Che	2025	A Bone Marrow-Mimetic Hydrogel Enables Dual-Phase Hemostasis and Vascularized Osteogenesis for Cranial Defects	10.1002/advs.75590	Include	Bioadhesive/interfacial biomaterial with direct bone-regeneration relevance and experimental validation.
ART-047	Qiuxia Huang	2025	ROS-responsive hydrogel for bone regeneration: Controlled dimethyl fumarate release to reduce inflammation and enhance osteogenesis	10.1016/j.actbio.2025.02.026	Exclude	Studies evaluating conventional bone cements, fixation devices, implant coatings, or scaffolds without a primary adhesive function or adhesion-related evaluation.
ART-048	Mengmeng Jin	2025	Polydopamine-Coated Copper-Doped Mesoporous Silica/Gelatin–Waterborne Polyurethane Composite: A Multifunctional GBR Membrane Bone Defect Repair	10.3390/jfb16040122	Exclude	Studies evaluating conventional bone cements, fixation devices, implant coatings, or scaffolds without a primary adhesive function or adhesion-related evaluation.
ART-049	Ziyang Bai	2025	Mussel-inspired bifunctional chimeric peptides macromolecules functionalize 3D-printed porous scaffolds for enhanced antimicrobial and osseointegration properties in bone defect repair	10.1016/j.ijbiomac.2025.142668	Exclude	Studies evaluating conventional bone cements, fixation devices, implant coatings, or scaffolds without a primary adhesive function or adhesion-related evaluation.
ART-050	Cheng Ji	2023	Mussel-inspired HA@TA-CS/SA biomimetic 3D printed scaffolds with antibacterial activity for bone repair	10.3389/fbioe.2023.1193605	Exclude	Studies evaluating conventional bone cements, fixation devices, implant coatings, or scaffolds without a primary adhesive function or adhesion-related evaluation.
ART-051	Alex P. Kleinfehn	2019	Modulating Bioglass Concentration in 3D Printed Poly(propylene fumarate) Scaffolds for Post-Printing Functionalization with Bioactive Functional Groups	10.1021/acs.biomac.9b00941	Exclude	Studies evaluating conventional bone cements, fixation devices, implant coatings, or scaffolds without a primary adhesive function or adhesion-related evaluation.
ART-052	Esensil Man Hia	2025	Synergistic effects of modified zinc oxide nanoparticle in a hybrid chitosan-gelatin hydrogel for bone regeneration	10.1016/j.ijbiomac.2025.144490	Exclude	Studies evaluating conventional bone cements, fixation devices, implant coatings, or scaffolds without a primary adhesive function or adhesion-related evaluation.
ART-053	Wuchao Ma	2023	Fish skin gelatin-based adhesive hydrogels loading cod peptides with osteogenic activity for bone tissue engineering	10.1016/j.colsurfa.2023.131695	Exclude	Studies evaluating conventional bone cements, fixation devices, implant coatings, or scaffolds without a primary adhesive function or adhesion-related evaluation.
ART-054	Haitao Cui	2016	Hierarchical Fabrication of Engineered Vascularized Bone Biphasic Constructs via Dual 3D Bioprinting: Integrating Regional Bioactive Factors into Architectural Design	10.1002/adhm.201600505	Exclude	Studies evaluating conventional bone cements, fixation devices, implant coatings, or scaffolds without a primary adhesive function or adhesion-related evaluation.
ART-055	Pei Feng	2021	Polydopamine modified polycaprolactone powder for fabrication bone scaffold owing intrinsic bioactivity	10.1016/j.jmrt.2021.09.137	Exclude	Studies evaluating conventional bone cements, fixation devices, implant coatings, or scaffolds without a primary adhesive function or adhesion-related evaluation.
ART-056	Jinshan Guo	2016	Synthesis and characterization of anti-bacterial and anti-fungal citrate-based mussel-inspired bioadhesives	10.1016/j.biomaterials.2016.01.069	Exclude	Studies focused exclusively on non-bone tissues or applications, including cartilage, skin, vascular, neural, tendon, periodontal, or soft-tissue regeneration without a direct bone-regenerative objective.
ART-057	Chuan Fu	2021	Mussel-inspired gold nanoparticle and plga/l-lysine-g-graphene oxide composite scaffolds for bone defect repair	10.2147/IJN.S328390	Exclude	Studies evaluating conventional bone cements, fixation devices, implant coatings, or scaffolds without a primary adhesive function or adhesion-related evaluation.
ART-058	Lu Han	2019	Mussel-inspired hybrid coating functionalized porous hydroxyapatite scaffolds for bone tissue regeneration	10.1016/j.colsurfb.2019.04.024	Exclude	Studies evaluating conventional bone cements, fixation devices, implant coatings, or scaffolds without a primary adhesive function or adhesion-related evaluation.
ART-059	Yiwen Chen	2025	ROS-responsive adaptive injectable hydrogel promoting inflammatory mastoid bone repair through efficient sterilization and regulating oxidative stress and macrophage phenotype	10.1016/j.mtbio.2025.101856	Include	Bioadhesive/interfacial biomaterial with direct bone-regeneration relevance and experimental validation.
ART-060	Guoqing Pan	2017	Biomimetic Design of Mussel-Derived Bioactive Peptides for Dual-Functionalization of Titanium-Based Biomaterials	10.1021/jacs.6b09770	Exclude	Studies evaluating conventional bone cements, fixation devices, implant coatings, or scaffolds without a primary adhesive function or adhesion-related evaluation.
ART-061	Xingchen Li	2024	Mussel-inspired antimicrobial hydrogel with cellulose nanocrystals/tannic acid modified silver nanoparticles for enhanced calvarial bone regeneration	10.1016/j.ijbiomac.2024.132419	Exclude	Studies evaluating conventional bone cements, fixation devices, implant coatings, or scaffolds without a primary adhesive function or adhesion-related evaluation.
ART-062	Kanwal Ilyas	2022	Surface Modification of 3D-Printed PCL/BG Composite Scaffolds via Mussel-Inspired Polydopamine and Effective Antibacterial Coatings for Biomedical Applications	10.3390/ma15238289	Exclude	Studies evaluating conventional bone cements, fixation devices, implant coatings, or scaffolds without a primary adhesive function or adhesion-related evaluation.
ART-063	Minhao Wu	2022	Mussel-inspired multifunctional surface through promoting osteogenesis and inhibiting osteoclastogenesis to facilitate bone regeneration	10.1038/s41536-022-00224-9	Exclude	Studies evaluating conventional bone cements, fixation devices, implant coatings, or scaffolds without a primary adhesive function or adhesion-related evaluation.
ART-064	A. Catarina Vale	2019	Antibacterial free-standing polysaccharide composite films inspired by the sea	10.1016/j.ijbiomac.2019.04.102	Exclude	Studies evaluating conventional bone cements, fixation devices, implant coatings, or scaffolds without a primary adhesive function or adhesion-related evaluation.
ART-065	Kai Jiang	2024	An immunomodulatory and osteogenic bacterial cellulose scaffold for bone	10.1016/j.ijbiomac.2024.136375	Exclude	Studies evaluating conventional bone cements, fixation devices, implant coatings,

			regeneration via regulating the immune microenvironment			or scaffolds without a primary adhesive function or adhesion-related evaluation.
ART-066	Mingjun Li	2020	Mussel-inspired multifunctional coating for bacterial infection prevention and osteogenic induction	10.1016/j.jmst.2020.08.011	Exclude	Studies evaluating conventional bone cements, fixation devices, implant coatings, or scaffolds without a primary adhesive function or adhesion-related evaluation.
ART-067	Guangyu Jian	2025	Enhanced sequential osteosarcoma therapy using a 3D-Printed bioceramic scaffold combined with 2D nanosheets via NIR-II photothermal-chemodynamic synergy	10.1016/j.bioactmat.2025.04.029	Exclude	Studies evaluating conventional bone cements, fixation devices, implant coatings, or scaffolds without a primary adhesive function or adhesion-related evaluation.
ART-068	Zhuoyue Chen	2018	Influence of Mussel-Derived Bioactive BMP-2-Decorated PLA on MSC Behavior in Vitro and Verification with Osteogenicity at Ectopic Sites in Vivo	10.1021/acsami.8b01547	Exclude	Studies evaluating conventional bone cements, fixation devices, implant coatings, or scaffolds without a primary adhesive function or adhesion-related evaluation.
ART-069	Kuan Zhang	2018	Bioinspired Surface Functionalization for Improving Osteogenesis of Electrospun Polycaprolactone Nanofibers	10.1021/acs.langmuir.8b03357	Exclude	Studies evaluating conventional bone cements, fixation devices, implant coatings, or scaffolds without a primary adhesive function or adhesion-related evaluation.
ART-070	Sumbul Mirza	2018	Synergistic combination of natural bioadhesive bael fruit gum and chitosan/nano-hydroxyapatite: A ternary bioactive nanohybrid for bone tissue engineering	10.1016/j.ijbiomac.2018.07.128	Exclude	Studies evaluating conventional bone cements, fixation devices, implant coatings, or scaffolds without a primary adhesive function or adhesion-related evaluation.
ART-071	Shuang Wang	2026	Friction-adaptive hydrogel coating for mechanical-immune synergy in ligament-to-bone integration	10.1016/j.biomaterials.2026.124003	Exclude	Studies focused exclusively on non-bone tissues or applications, including cartilage, skin, vascular, neural, tendon, periodontal, or soft-tissue regeneration without a direct bone-regenerative objective.
ART-072	Huiyong Xu	2018	Mussel-inspired dual-functional PEG hydrogel inducing mineralization and inhibiting infection in maxillary bone reconstruction	10.1016/j.msec.2018.04.066	Exclude	Studies evaluating conventional bone cements, fixation devices, implant coatings, or scaffolds without a primary adhesive function or adhesion-related evaluation.
ART-073	Shiwen Zhang	2016	Mussel-inspired alginate gel promoting the osteogenic differentiation of mesenchymal stem cells and anti-infection	10.1016/j.msec.2016.06.044	Exclude	Studies evaluating conventional bone cements, fixation devices, implant coatings, or scaffolds without a primary adhesive function or adhesion-related evaluation.
ART-074	Jiamin Zhou	2024	ZIF-8-Modified Multifunctional Hydrogel Loading siRNA and DOX for Postoperative Therapy of Maxillofacial Osteosarcoma and Bone Repair	10.1021/acsami.4c21331	Exclude	Studies evaluating conventional bone cements, fixation devices, implant coatings, or scaffolds without a primary adhesive function or adhesion-related evaluation.
ART-075	Yilin Mao	2022	Wet-adhesive materials of oral and maxillofacial region: From design to application	10.1016/j.cclet.2022.04.059	Exclude	Review articles, systematic reviews, meta-analyses, editorials, letters to the editor, commentaries, conference abstracts, book chapters, dissertations, patents, and expert opinions.
ART-076	Qian Xu	2022	Mussel-inspired bioactive 3D-printable poly(styrene-butadiene-styrene) and the in vitro assessment of its potential as cranioplasty implants	10.1039/d2tb00419d	Exclude	Studies evaluating conventional bone cements, fixation devices, implant coatings, or scaffolds without a primary adhesive function or adhesion-related evaluation.
ART-077	Esensil Man Hia	2024	Construction of a PEGDA/chitosan hydrogel incorporating mineralized copper-doped mesoporous silica nanospheres for accelerated bone regeneration	10.1016/j.ijbiomac.2024.130218	Exclude	Studies evaluating conventional bone cements, fixation devices, implant coatings, or scaffolds without a primary adhesive function or adhesion-related evaluation.
ART-078	Kullapop Suttiat	2022	Preparation and Characterization of Porous Poly(Lactic Acid)/Poly(Butylene Adipate-Co-Terephthalate) (PLA/PBAT) Scaffold with Polydopamine-Assisted Biomineralization for Bone Regeneration	10.3390/ma15217756	Exclude	Studies evaluating conventional bone cements, fixation devices, implant coatings, or scaffolds without a primary adhesive function or adhesion-related evaluation.
ART-079	Jingyang Kang	2025	Mussel-inspired deposition of Cu and Zn on PDA-coated titanium for synergistic antibacterial and osteogenic effects	10.1016/j.colsurfa.2025.137828	Exclude	Studies evaluating conventional bone cements, fixation devices, implant coatings, or scaffolds without a primary adhesive function or adhesion-related evaluation.
ART-080	Lu Han	2017	Mussel-inspired graphene oxide nanosheet-enwrapped Ti scaffolds with drug-encapsulated gelatin microspheres for bone regeneration	10.1039/c7bm01060e	Exclude	Studies evaluating conventional bone cements, fixation devices, implant coatings, or scaffolds without a primary adhesive function or adhesion-related evaluation.
ART-081	Heqin Huang	2016	Bioactive Nanocomposite Poly (Ethylene Glycol) Hydrogels Crosslinked by Multifunctional Layered Double Hydroxides Nanocrosslinkers	10.1002/mabi.201600054	Include	Bioadhesive/interfacial biomaterial with direct bone-regeneration relevance and experimental validation.
ART-082	Alexander J. Steeves	2019	Elucidating structure-function relationships governing the interfacial response of human mesenchymal stem cells to polydopamine coatings	10.1039/c9tb02188d	Exclude	Studies evaluating conventional bone cements, fixation devices, implant coatings, or scaffolds without a primary adhesive function or adhesion-related evaluation.
ART-083	Jussi Sarin	2016	Compression properties and dissolution of bioactive glass S53P4 and n-butyl-2 cyanoacrylate tissue adhesive-composite	10.3233/BME-161596	Exclude	Studies evaluating conventional bone cements, fixation devices, implant coatings, or scaffolds without a primary adhesive function or adhesion-related evaluation.
ART-084	Mobina Bakhshyani	2021	Development of a high-performance PVA/DOPA bone adhesive incorporated with bioactive glass and hydroxyapatite particles for highly comminuted bone fractures	10.1080/25740881.2021.1995419	Exclude	Studies evaluating conventional bone cements, fixation devices, implant coatings, or scaffolds without a primary adhesive function or adhesion-related evaluation.
ART-085	Fu-Chen Kung	2015	Osteogenesis of human adipose-derived stem cells on poly(dopamine)-coated electrospun poly(lactic acid) fiber mats	10.1016/j.msec.2015.08.009	Exclude	Studies evaluating conventional bone cements, fixation devices, implant coatings,

						or scaffolds without a primary adhesive function or adhesion-related evaluation.
ART-086	Weitong Chen	2022	A hybrid coating of polydopamine and nano-hydroxyapatite enhances surface properties of 3D printed poly(lactic-co-glycolic acid) scaffolds	10.1007/s10853-022-07442-y	Exclude	Studies evaluating conventional bone cements, fixation devices, implant coatings, or scaffolds without a primary adhesive function or adhesion-related evaluation.
ART-087	Sang Jin Lee	2016	Surface modification of 3D-printed porous scaffolds via mussel-inspired polydopamine and effective immobilization of rhBMP-2 to promote osteogenic differentiation for bone tissue engineering	10.1016/j.actbio.2016.02.006	Exclude	Studies evaluating conventional bone cements, fixation devices, implant coatings, or scaffolds without a primary adhesive function or adhesion-related evaluation.
ART-088	Daniela N Céspedes-Valenzuela	2022	Preparation and Characterization of an Injectable and Photo-Responsive Chitosan Methacrylate/Graphene Oxide Hydrogel: Potential Applications in Bone Tissue Adhesion and Repair	10.3390/polym14010126	Include	Bioadhesive/interfacial biomaterial with direct bone-regeneration relevance and experimental validation.
ART-089	Yong Xu	2018	Interfacial reinforcement in a poly-l-lactic acid/mesoporous bioactive glass scaffold via polydopamine	10.1016/j.colsurfb.2018.05.065	Exclude	Studies not reporting any evaluation of adhesive performance when adhesion is a claimed functional property.
ART-090	Xiao-Tong Su	2023	Effects of methoxy polyethylene glycol-polycaprolactone block copolymers and silica nanoparticles on the mechanical, tissue affinity and antibacterial properties of n-butyl cyanoacrylate adhesive	10.1002/app.54342	Exclude	Studies focused exclusively on non-bone tissues or applications, including cartilage, skin, vascular, neural, tendon, periodontal, or soft-tissue regeneration without a direct bone-regenerative objective.
ART-091	Huan Zhao	2018	Mussel-Inspired Peptide Coatings on Titanium Implant to Improve Osseointegration in Osteoporotic Condition	10.1021/acsbiomaterials.8b00261	Exclude	Studies not reporting any evaluation of adhesive performance when adhesion is a claimed functional property.
