# Supplementary material for: Natural and Hybrid Bioadhesive Polymeric Platforms for Bone Regeneration: A PRISMA-Guided Systematic Review of Design Strategies and Translational Challenges
Source: Polymers (Basel). 2026 Sep 17;18(18):2266. doi: 10.3390/polym18182266 (PMC13611712; doi:10.3390/polym18182266)
Supplement: Supplementary file 1 [file polymers-18-02266-s001.zip › Supplementary File S3 PRISMA_2020 Checklist.pdf]

**PRISMA 2020 Checklist**

**Manuscript title:** *Natural and Hybrid Bioadhesive Polymeric Platforms for Bone Regeneration: A PRISMA-Guided Systematic Review of Design Strategies and Translational Challenges*
**Journal:** *Polymers*
**Article type:** Systematic Review

| Section and topic | Item No. | PRISMA 2020 checklist item | Location where item is reported |
|---|---|---|---|
| **Title** | 1 | Identify the report as a systematic review. | Title page. The title explicitly identifies the manuscript as a PRISMA-guided systematic review. |
| **Abstract** | 2 | See the PRISMA 2020 for Abstracts checklist. | Abstract. The abstract summarizes the background, objective, PRISMA-guided methodology, number of included studies, main findings, and translational challenges. |
| **Introduction — Rationale** | 3 | Describe the rationale for the review in the context of existing knowledge. | Introduction. The rationale is provided through the discussion of bone regeneration challenges, limitations of conventional biomaterials, and the emerging role of natural and hybrid bioadhesive polymeric platforms. |
| **Introduction — Objectives** | 4 | Provide an explicit statement of the objective(s) or question(s) the review addresses. | End of Introduction and Section 2.1. The review question and eligibility criteria are structured using the PICOC framework. |
| **Methods — Eligibility criteria** | 5 | Specify the inclusion and exclusion criteria for the review and how studies were grouped for the syntheses. | Section 2.1 and eligibility criteria subsection. The PICOC framework defines population, intervention, comparison, outcome, and context. Studies were grouped according to biomaterial platform, adhesive mechanism, regenerative function, and translational complexity. |
| **Methods — Information sources** | 6 | Specify all databases, registers, websites, organisations, reference lists, and other sources searched or consulted to identify studies. Specify the date when each source was last searched or consulted. | Search Strategy section and Supplementary Material, Appendix A. The databases searched were PubMed/MEDLINE, Web of Science, Scopus, and IEEE Xplore. The final search was performed on 11 May 2026. |
| **Methods — Search strategy** | 7 | Present the full search strategies for all databases, registers, and websites, including any filters and limits used. | Supplementary Material, Appendix A. Full database-specific search equations are provided for PubMed/MEDLINE, Web of Science, Scopus, and IEEE Xplore. The uploaded supplementary file explicitly includes these equations and the final search date. |
| **Methods — Selection process** | 8 | Specify the methods used to decide whether a study met the inclusion criteria, including how many reviewers screened each record and report retrieved, whether they worked independently, and how disagreements were resolved. | Methods section, study selection subsection. Records were identified, duplicates removed, titles and abstracts screened, and full texts assessed according to predefined eligibility criteria. |

| Section and topic | Item No. | PRISMA 2020 checklist item | Location where item is reported |
|---|---|---|---|
| **Methods — Data collection process** | 9 | Specify the methods used to collect data from reports, including how many reviewers collected data, whether they worked independently, and any processes for obtaining or confirming data from study investigators. | Methods section, data extraction subsection. Relevant information was extracted from included studies using a structured review matrix. |
| **Methods — Data items** | 10a | List and define all outcomes for which data were sought. Specify whether all results compatible with each outcome domain in each study were sought. | Methods section. The outcomes included polymeric matrix, adhesive mechanism, crosslinking strategy, bioactive components, regenerative function, biological performance, experimental model, and translational limitations. |
| **Methods — Data items** | 10b | List and define all other variables for which data were sought. | Methods section and Supplementary Tables S1 and S2. Additional variables included developmental category, functional complexity, predominant bioadhesion mechanism, and translational relevance. Supplementary Tables S1 and S2 summarize these extracted variables. |
| **Methods — Study risk of bias assessment** | 11 | Specify the methods used to assess risk of bias in the included studies, including details of the tool(s) used, how many reviewers assessed each study, and whether they worked independently. | Methods section. A formal clinical risk-of-bias tool was not applied because the review focused on heterogeneous experimental biomaterial studies rather than randomized clinical trials. Methodological limitations and translational weaknesses were assessed narratively. |
| **Methods — Effect measures** | 12 | Specify for each outcome the effect measure(s) used in the synthesis or presentation of results. | Not applicable. No meta-analysis or pooled quantitative effect estimate was performed because of the heterogeneity of biomaterial compositions, testing methods, experimental models, and reported outcomes. |
| **Methods — Synthesis methods** | 13a | Describe the processes used to decide which studies were eligible for each synthesis. | Methods section. Included studies were synthesized qualitatively according to polymeric platform, adhesive mechanism, regenerative function, and level of functional/translational complexity. |
| **Methods — Synthesis methods** | 13b | Describe any methods required to prepare the data for presentation or synthesis. | Methods section. Data were extracted, categorized, and organized into comparative tables and narrative thematic sections. |
| **Methods — Synthesis methods** | 13c | Describe any methods used to tabulate or visually display results of individual studies and syntheses. | Results section, Figures 3 and 4, and Supplementary Tables S1 and S2. Results were summarized using temporal/geographical analysis, developmental classification, and bioadhesion mechanism tables. |
| **Methods — Synthesis methods** | 13d | Describe any methods used to synthesize results and provide a rationale for the choice. | Methods section. A narrative synthesis was used because of the methodological and experimental heterogeneity of the included biomaterial studies. |
| **Methods — Synthesis methods** | 13e | Describe any methods used to explore possible causes of heterogeneity among study results. | Discussion section. Heterogeneity was explored narratively in relation to polymeric composition, |

| Section and topic | Item No. | PRISMA 2020 checklist item | Location where item is reported |
| --- | --- | --- | --- |
| | | | adhesive chemistry, biological model, defect type, testing protocol, and translational readiness. |
| **Methods — Synthesis methods** | 13f | Describe any sensitivity analyses conducted to assess robustness of the synthesized results. | Not applicable. No quantitative sensitivity analysis was performed because no meta-analysis was conducted. |
| **Methods — Reporting bias assessment** | 14 | Describe any methods used to assess risk of bias due to missing results in a synthesis. | Not applicable. Formal reporting bias assessment was not performed because the review did not include meta-analysis or pooled quantitative synthesis. Potential reporting limitations are discussed narratively. |
| **Methods — Certainty assessment** | 15 | Describe any methods used to assess certainty or confidence in the body of evidence for an outcome. | Discussion section. Certainty was not graded using GRADE because the review focused on preclinical and experimental biomaterial studies. Confidence in the evidence was discussed narratively considering experimental model, biological validation, adhesion testing, and translational limitations. |
| **Results — Study selection** | 16a | Describe the results of the search and selection process, from the number of records identified to the number of studies included, ideally using a flow diagram. | Results section and Figure 2. The PRISMA 2020 flow diagram reports the identification, screening, eligibility assessment, and final inclusion of studies. |
| **Results — Study selection** | 16b | Cite studies that might appear to meet the inclusion criteria but were excluded, and explain why they were excluded. | Methods/Results section. Full-text exclusions were summarized according to predefined reasons in the study selection process and PRISMA flow diagram. |
| **Results — Study characteristics** | 17 | Cite each included study and present its characteristics. | Results section and Supplementary Table S1. The 38 included studies are classified according to developmental category and functional/translational complexity. |
| **Results — Risk of bias in studies** | 18 | Present assessments of risk of bias for each included study. | Not applicable. A formal risk-of-bias assessment was not performed due to the experimental and heterogeneous nature of the included biomaterial studies. Methodological and translational limitations are discussed narratively. |
| **Results — Results of individual studies** | 19 | For all outcomes, present for each study summary statistics and effect estimates, where applicable. | Results section and Supplementary Tables S1 and S2. The review presents qualitative outcomes related to adhesive mechanism, regenerative function, biological performance, and developmental classification. |
| **Results — Results of syntheses** | 20a | For each synthesis, briefly summarise the characteristics and risk of bias among contributing studies. | Results and Discussion sections. The qualitative synthesis summarizes study characteristics according to polymeric platform, bioadhesion mechanism, regenerative function, and translational complexity. |

| Section and topic | Item No. | PRISMA 2020 checklist item | Location where item is reported |
|---|---|---|---|
| **Results — Results of syntheses** | 20b | Present results of all statistical syntheses conducted. | Not applicable. No statistical synthesis or meta-analysis was performed. |
| **Results — Results of syntheses** | 20c | Present results of all investigations of possible causes of heterogeneity among study results. | Discussion section. Heterogeneity is discussed in relation to material composition, crosslinking chemistry, adhesion testing, biological models, and translational maturity. |
| **Results — Results of syntheses** | 20d | Present results of all sensitivity analyses conducted to assess robustness of the synthesized results. | Not applicable. No quantitative sensitivity analysis was conducted. |
| **Results — Reporting biases** | 21 | Present assessments of risk of bias due to missing results for each synthesis assessed. | Not applicable. Formal reporting bias assessment was not performed. Limitations related to incomplete reporting and lack of standardized testing are discussed narratively. |
| **Results — Certainty of evidence** | 22 | Present assessments of certainty or confidence in the body of evidence for each outcome assessed. | Discussion section. The level of confidence in the evidence is discussed narratively, emphasizing the predominance of preclinical models, heterogeneity of testing methods, and limited translational validation. |
| **Discussion — Discussion** | 23a | Provide a general interpretation of the results in the context of other evidence. | Discussion section. The manuscript interprets the evolution from conventional adhesive systems toward multifunctional and smart regenerative bioadhesive platforms. |
| **Discussion — Discussion** | 23b | Discuss any limitations of the evidence included in the review. | Discussion section. Limitations include heterogeneous adhesion testing, limited load-bearing models, variability in animal models, and insufficient translational validation. |
| **Discussion — Discussion** | 23c | Discuss any limitations of the review processes used. | Discussion section. Limitations of the review process are addressed, including restriction to English-language original research and the narrative nature of the synthesis. |
| **Discussion — Discussion** | 23d | Discuss implications of the results for practice, policy, and future research. | Discussion and Conclusions sections. The manuscript discusses implications for biomaterial design, standardization of bone-specific adhesion testing, preclinical validation, manufacturing, regulation, and translational development. |
| **Other information — Registration and protocol** | 24a | Provide registration information for the review, including register name and registration number, or state that the review was not registered. | Section 2.1. The manuscript states that the review protocol was not prospectively registered. |
| **Other information — Registration and protocol** | 24b | Indicate where the review protocol can be accessed, or state that a protocol was not prepared. | Section 2.1. The review protocol was not prospectively registered. |
| **Other information — Registration and protocol** | 24c | Describe and explain any amendments to information | Not applicable. No prospective registration or registered protocol was available. |

| Section and topic | Item No. | PRISMA 2020 checklist item | Location where item is reported |
|---|---|---|---|
| | | provided at registration or in the protocol. | |
| **Other information — Support** | 25 | Describe sources of financial or non-financial support for the review, and the role of the funders or sponsors. | Funding section. The manuscript reports institutional and project-related funding information. |
| **Other information — Competing interests** | 26 | Declare any competing interests of review authors. | Conflicts of Interest section. The authors declare no conflicts of interest. |
| **Other information — Availability of data, code, and other materials** | 27 | Report which data, code, and other materials are publicly available and where they can be found. | Data Availability Statement and Supplementary Material. The original contributions are included in the article and supplementary material. Further inquiries can be directed to the corresponding author(s). Supplementary Material includes Appendix A, Table S1, and Table S2. |

**Note:** Items related to quantitative synthesis, effect measures, meta-analysis, sensitivity analysis, and formal reporting bias assessment were considered not applicable because this systematic review provides a qualitative narrative synthesis of heterogeneous experimental biomaterial studies rather than a pooled quantitative meta-analysis.
